# Characterization of zebrafish GABA_A_ receptor subunits

**DOI:** 10.1038/s41598-021-84646-3

**Published:** 2021-03-18

**Authors:** Kenichiro Sadamitsu, Leona Shigemitsu, Marina Suzuki, Daishi Ito, Makoto Kashima, Hiromi Hirata

**Affiliations:** Department of Chemistry and Biological Science, College of Science and Engineering, Aoyama Gakuin University, Sagamihara, 252-5258 Japan

**Keywords:** Neuronal physiology, Synaptic transmission

## Abstract

γ-Aminobutyric acid (GABA), the major inhibitory neurotransmitter in the central nervous system, exerts its effect through the activation of GABA receptors. GABA_A_ receptors are ligand-gated chloride channels composed of five subunit proteins. Mammals have 19 different GABA_A_ receptor subunits (α1–6, β1–3, γ1–3, δ, ε, π, θ, and ρ1–3), the physiological properties of which have been assayed by electrophysiology. However, the evolutionary conservation of the physiological characteristics of diverged GABA_A_ receptor subunits remains unclear. Zebrafish have 23 subunits (α1, α2a, α2b, α3–5, α6a, α6b, β1–4, γ1–3, δ, π, ζ, ρ1, ρ2a, ρ2b, ρ3a, and ρ3b), but the electrophysiological properties of these subunits have not been explored. In this study, we cloned the coding sequences for zebrafish GABA_A_ receptor subunits and investigated their expression patterns in larval zebrafish by whole-mount in situ hybridization. We also performed electrophysiological recordings of GABA-evoked currents from *Xenopus* oocytes injected with one or multiple zebrafish GABA_A_ receptor subunit cRNAs and calculated the half-maximal effective concentrations (EC50s) for each. Our results revealed the spatial expressions and electrophysiological GABA sensitivities of zebrafish GABA_A_ receptors, suggesting that the properties of GABA_A_ receptor subunits are conserved among vertebrates.

## Introduction

γ-Aminobutyric acid (GABA), the major inhibitory neurotransmitter in the central nervous system of vertebrates, controls the excitability of neural networks mainly through GABA_A_ receptors^1^. The GABA_A_ receptor mediates two types of inhibition, known as phasic and tonic inhibition^2^. Phasic inhibition occurs at postsynaptic sites, where the GABA concentration transiently rises to more than 1 mM during synaptic transmission^3^, while tonic inhibition occurs at extrasynaptic sites, where the concentration of spillover GABA increases to ~ 0.5 µM^4,5^. Regardless of synaptic or extrasynaptic sites, GABA_A_ receptors comprise five subunits forming Cl^–^conducting channels. Each subunit has a large extracellular N-terminal domain that contributes to GABA binding, followed by four transmembrane domains, with the second one forming the channel pore. Mammals have 19 different GABA_A_ receptor subunits (α1–6, β1–3, γ1–3, δ, ε, π, θ, and ρ1–3). Although this diversity may allow for numerous possible combinations of subunits, most GABA_A_ receptors are composed of two α, two β, and one γ subunit^6^. In fact, experimental evidence of native GABA_A_ receptors suggests that there are fewer than 20 receptor subtypes, with the major synaptic GABA_A_ receptor combinations being α1β2γ2, α1β3γ2, α2β3γ2, and α3β3γ2^7,8^. The extrasynaptic GABA_A_ receptors appear to contain specific subunits such as α4, α5, α6, and δ, forming α4β3δ, α5β3γ2, and α6β3δ^2,7^. The other subunits—ε, π, and θ—also assemble with α and β subunits and are located at extrasynaptic sites^2^. The ρ subunits form homopentameric GABA_A_ receptors that are predominantly expressed in the retina^9^. The β3 subunit can also form homopentameric channels that are activated by the anesthetic agent etomidate and a high concentration (~ 10 mM) of GABA in *Xenopus* oocytes^10^. While the physiological properties of GABA_A_ receptor isoforms in mammals have been addressed, the evolutionary conservation and physiological significance of diverged subunits in vertebrates remain largely unclear.

GABA_A_ receptors have also been studied in zebrafish, a vertebrate model that offers advantages such as the production of many offspring, fast embryonic development, optical transparency during embryogenesis, rapid acquisition of locomotor behaviors, and the ease of pharmacological treatment^11^. Cocco and colleagues identified 23 putative GABA_A_ receptor subunits (eight α, four β, three γ, one δ, one π, one ζ, and five ρ) in the zebrafish genome^12^. They also investigated the transcript levels of GABA_A_ receptor subunits in the adult zebrafish brain using reverse transcription-polymerase chain reaction (RT-qPCR). Another recent study assayed the spatial expression patterns of eight GABA_A_ receptor α subunits in zebrafish embryos by in situ hybridization and showed that most α subunits are expressed during embryogenesis^13^. Several loss-of-function studies have revealed the physiological function of GABA_A_ receptors in zebrafish. Antisense morpholino-mediated knockdown of the α1 subunit caused reduced spontaneous locomotor activity in larvae at 5 days post-fertilization (dpf)^14^, while CRISPR/Cas9-mediated knockout of α1 caused seizure phenotypes in juveniles at 35 dpf^15^. Knockdown of the α2 subunit perturbed the expression of the proneural gene *neurod* and a GABA-synthesizing enzyme gene *gad1b* within 1 day of development^16^. Zebrafish larvae lacking the β3 subunit showed reduced sensitivity to anesthetic drugs such as etomidate and propofol^17^. Patch-clamp recordings of GABA_A_ receptor-mediated miniature inhibitory postsynaptic currents from zebrafish Mauthner cells revealed three different types of gating kinetics, suggesting that zebrafish also have multiple GABA_A_ receptor subtypes comprising different subunit combinations^18^. However, the electrophysiological characteristics of the zebrafish GABA_A_ receptor subunit have not yet been explored.

In this study, we performed phylogenetic analysis and cloned cDNAs for zebrafish GABA_A_ receptor subunits. Our whole-mount in situ hybridization revealed the spatial expression patterns of GABA_A_ receptor subunit genes in 5 dpf larvae. We also assayed GABA-mediated gating of zebrafish GABA_A_ receptors composed of various combinations of receptor subunits in *Xenopus* oocytes. These attempts provide useful information on the spatial expressions and electrophysiological GABA sensitivities of zebrafish GABA_A_ receptors and suggest that the properties of GABA_A_ receptor subunits are conserved among vertebrates.

## Results

### Phylogenetic analysis and cloning of zebrafish GABA_A_ receptor subunits

**Figure 1:**
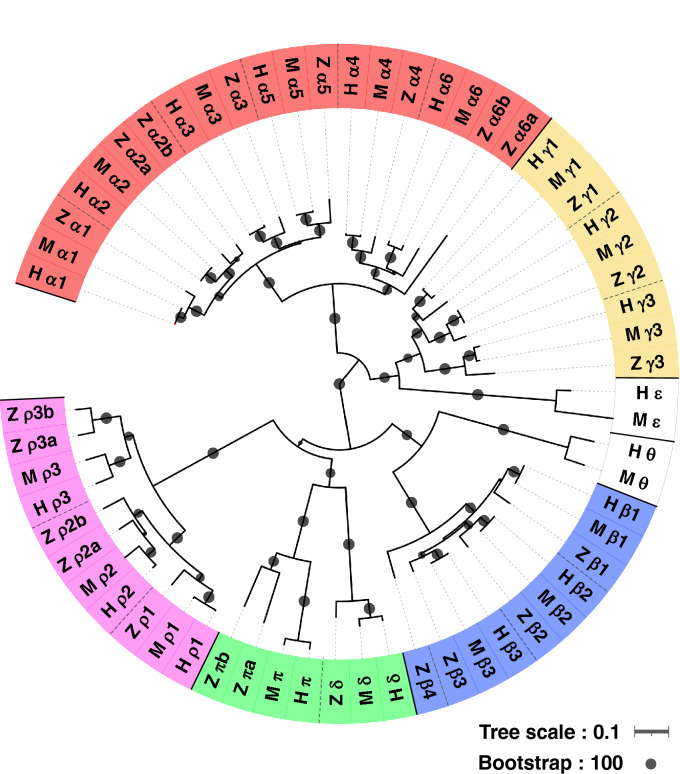
A phylogenetic tree of GABA_A_ receptor subunits. Amino acid sequences of GABA_A_ receptor subunits from humans, mice, and zebrafish were used to create a phylogenetic tree. This phylogenetic tree detected the subfamilies of α, γ/ ε, β/ θ, δ/π, and ρ. H: human; M: mouse; Z: zebrafish.

Nineteen GABA_A_ receptor subunits/genes have been identified in mammals (α1–6/*gabra1–6*, β1–3/*gabrb1–3*, γ1–3/*gabrg1–3*, δ/*gabrd*, ε/*gabre*, π/*gabrp*, θ/*gabrq*, and ρ1–3/*gabrr1–3*)^19^. Previous searches for GABA_A_ receptor subunits in the zebrafish genome database have suggested 23 GABA_A_ receptor subunits/genes comprising eight α (α1/*gabra1*, α2a/*gabra2a*, α2b/*gabra2b*, α3–5/*gabra3–5*, α6a/*gabra6a*, and α6b/*gabra6b*), four β (β1–4/*gabrb1–4*), three γ (γ1–3/*gabrg1–3*), one δ/*gabrd*, one π /*gabrp*, and five ρ (ρ1/*gabrr1*, ρ2a/*gabrr2a*, ρ2b/*gabrr2b*, ρ3a/*gabrr3a*, ρ3b/*gabrr3b*) as well as additional ζ/*gabrz* subunits, but neither ε nor θ subunits^12,13^. Some subunits that have a or b at the end of the subunit/gene name are paralogs generated by a suspected duplication of the whole genome during fish evolution^20^. We recapitulated in silico analysis using human and mouse GABA_A_ receptor protein sequences as queries to obtain zebrafish GABA_A_ receptor protein sequences. We successfully cloned cDNAs for all zebrafish GABA_A_ receptor subunits except for α2b from an RNA mixture extracted from a pool of 1–5 dpf zebrafish embryos/larvae. The previously annotated zebrafish α2b subunit (XP_017214538.1) showed 86% amino acid identity to the zebrafish α2a subunit in the N-terminus (exons 1–8) and only 10% identity in the C-terminus (exon 9). Therefore, the α2b information has been removed from the National Center for Biotechnology Information (NCBI) annotation as it was presumably caused by an incorrect annotation of the last exon. We then searched for another exon encoding the C-terminus of α2b in the genome database using the C-terminus protein sequence of zebrafish α2a as a query and identified the other last exon encoding a possible α2b C-terminus that showed 76% amino acid identity to zebrafish α2a. We successfully cloned the intact coding sequence of this newly annotated subunit and named zebrafish GABA_A_ receptor α2b subunit (LC596832), which differed from the previous annotation only in the last exon. We then updated the phylogenetic tree of human, mouse, and zebrafish GABA_A_ receptor subunits (Fig. 1). Our amino acid alignments of the GABA_A_ receptor subunits showed that each subunit is conserved among vertebrates, especially in four transmembrane domains (Supplementary Figs. 1–17; Supplementary Table 1). We also confirmed that the ζ subunit, which is found in zebrafish but not in mammals, belongs to the π subfamily, with the highest similarity to the zebrafish π subunit, indicating that the ζ subunit is a paralog of the π subunit. Thus, we suggest renaming the π and ζ subunits to πa and πb subunits, respectively. We hereafter refer to π and ζ as π/πa and ζ/πb, respectively.

### Spatial expression of zebrafish GABA_A_ receptor genes

**Figure 2:**
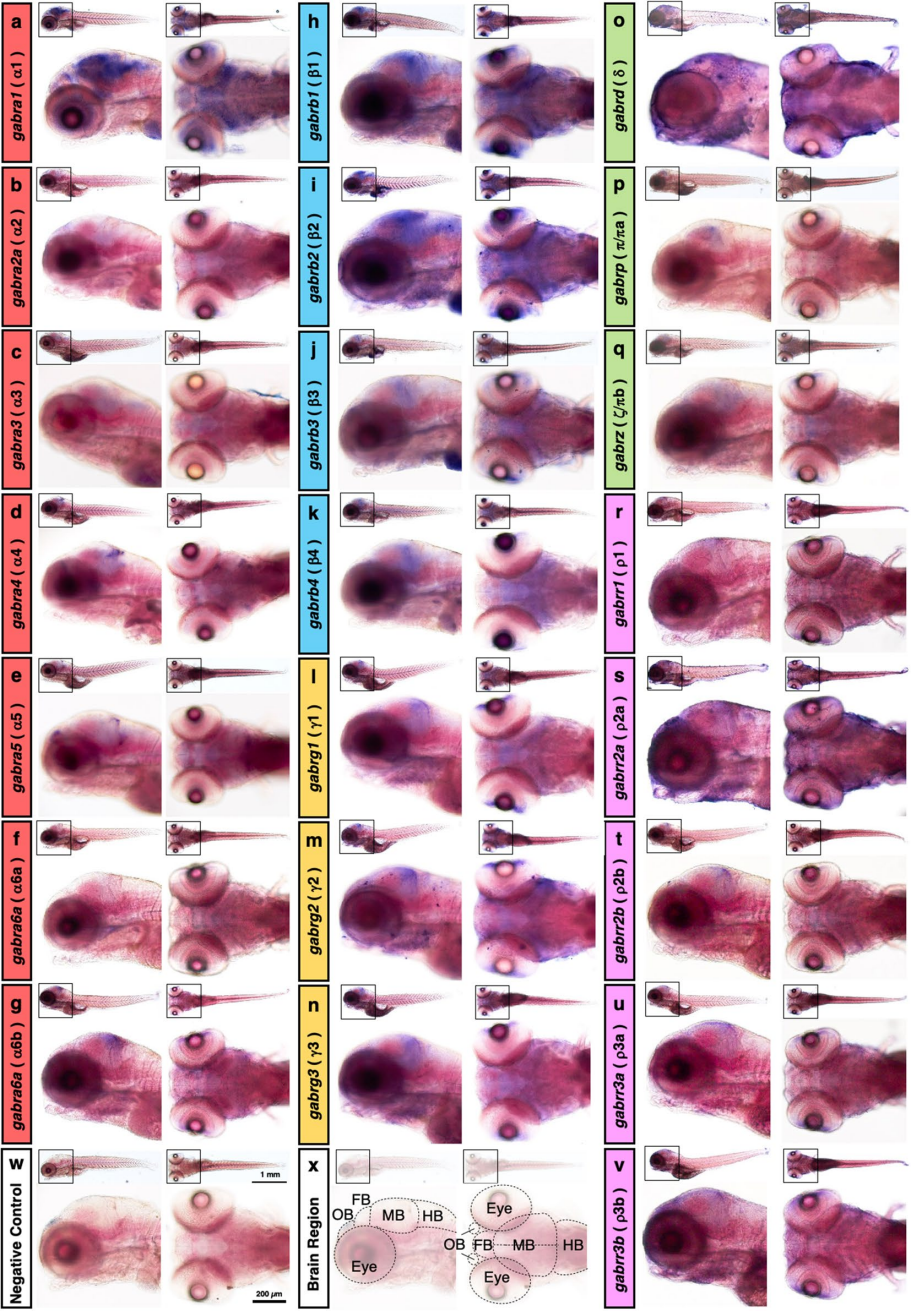
Spatial expression of zebrafish GABA_A_ receptor subunits. Whole-mount in situ hybridization of 5 dpf zebrafish larvae using antisense probes for *gabra1* (**a**), *gabra2a* (**b**), *gabra3* (**c**), *gabra4* (**d**), *gabra5* (**e**), *gabra6a* (**f**), *gabra6b* (**g**), *gabrb1* (**h**), *gabrb2* (**i**), *gabrb3* (**j**), *gabrb4* (**k**), *gabrg1* (**l**), *gabrg2* (**m**), *gabrg3* (**n**), *gabrd* (**o**), *gabrp* (**p**), *gabrz* (**q**), *gabrr1* (**r**), *gabrr2a* (**s**), *gabrr2b* (**t**), *gabrr3a* (**u**), and *gabrr3b* (**v**). Negative control without probe showing no signals (**w**). Regions of the olfactory bulb, forebrain, midbrain, hindbrain, and eye are indicated in the image (**x**). Each labeling image is composed of a whole lateral view (left top), a whole dorsal view (right top), a magnified lateral view of the head region (left bottom), and a magnified dorsal view of the head region (right bottom). The scale bars in the whole and magnified views are 1 mm and 200 μm, respectively. OB: olfactory bulb; FB: forebrain; MB: midbrain; HB: hindbrain.

A previous RT-PCR analysis described the expression of some, but not all, GABA_A_ receptor genes in the brain and eye of adult zebrafish^12^. Another whole-mount in situ hybridization study reported the spatial expression patterns of eight subunit genes in zebrafish embryos at 1, 2, and 4 dpf^13^. Since zebrafish larvae with a defect in the α1 gene showed seizure-like motor activity as early as 4 dpf^15^ and GABA_A_ receptor antagonist-induced zebrafish seizure can be assayed at 7 dpf^21^, we investigated the spatial expression of GABA_A_ receptor subunit genes by whole-mount in situ hybridization in zebrafish larvae at 5 dpf, when the deficiency of GABA_A_ receptor is likely correlated with seizure. The α1 gene was predominantly expressed in the forebrain, midbrain, hindbrain, and eye, while the other subunit genes were expressed by different but restricted patterns in the olfactory bulb, forebrain, midbrain, and eye at low levels (Fig. 2a–g,w,x). Probes for β subunits showed broad labeling in the whole brain (Fig. 2h–k). Expression of all three γ subunit genes was observed in broad brain regions including the olfactory bulb, forebrain, midbrain, hindbrain, and eye (Fig. 2l–n). The δ and ζ/πb genes were also expressed in the broad brain regions, while the π/πa gene was expressed in the restricted pattern in the midbrain and eye (Fig. 2o–q). Among the five ρ subunits, the ρ2a gene was predominantly expressed in the olfactory bulb, forebrain, midbrain, hindbrain, and eye (Fig. 2s). Expression of the other ρ subunit genes was also observed at low levels in the broad brain regions (Fig. 2r–v). We have summarized the spatial expressions with staining intensities indicated by +++, ++, or + in Table 1. These different but overlapping expressions of GABA_A_ receptor subunit genes suggest the formation of various GABA_A_ receptor subtypes comprising different subunit combinations.

### GABA concentration–response of zebrafish GABA_A_ receptor subtypes

**Figure 3:**
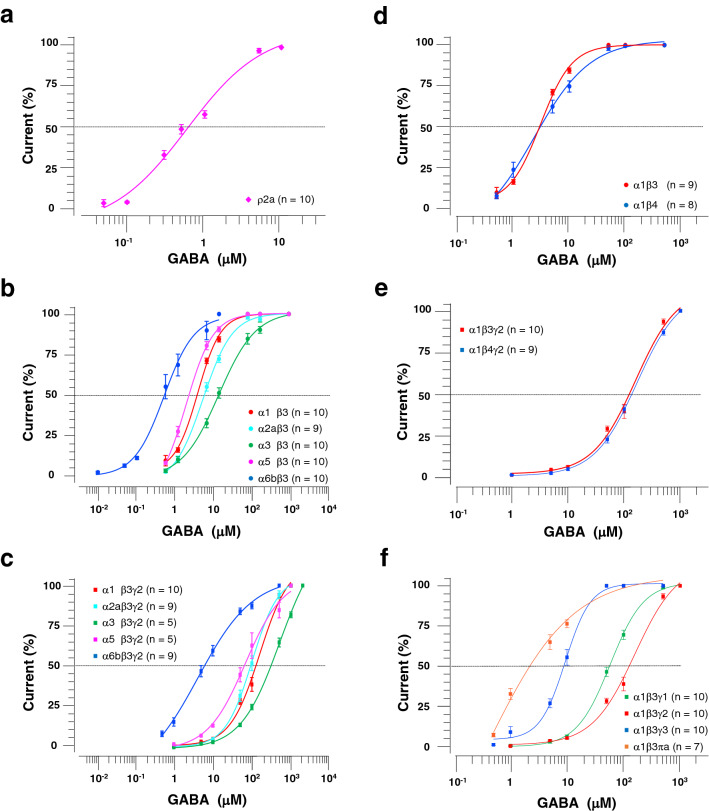
Electrophysiological sensitivities of zebrafish GABA_A_ receptors to GABA. (**a**) Cumulative dose–response relationship of GABA-evoked currents recorded from *Xenopus* oocytes expressing the ρ2a subunit. (**b**) Cumulative dose–response relationship of GABA currents by the recombinant expression of the α subunit with the β3 subunit. (**c**) Cumulative dose–response relationship of GABA currents by the expression of the α subunit with the β3 and γ2 subunits. (**d**) Cumulative dose–response relationship of GABA currents by the expression of the β subunit with the α1 subunit. (**e**) Cumulative dose–response relationship of GABA currents by the expression of the β subunit with the α1 and γ2 subunits. (**f**) Cumulative dose–response relationship of GABA currents by the expression of γ or π subunits with α1 and β3 subunits. The amplitude of each GABA-evoked response was normalized to the maximally evoked current for each oocyte.

To assess the electrophysiological properties of zebrafish GABA_A_ receptor subunits, we employed two-electrode voltage-clamp recordings and recorded GABA-evoked currents from *Xenopus* oocytes expressing single or multiple GABA_A_ receptor subunits. We first recorded GABA currents from oocytes injected with one type of subunit cRNAs. The expression of single α, γ, δ, π/πa, or ζ/πb subunits did not generate GABA-evoked currents at any GABA concentration, while that of either single β subunit yielded small currents (β1: 27.5 ± 10.7 nA, n = 4; β2: 29.4 ± 6.3 nA, n = 5; β3: 81.7 ± 7.5 nA, n = 5; β4: 28.3 ± 3.6 nA, n = 5) only in the presence of GABA at 10 mM, which is a non-physiological concentration at both synaptic and extrasynaptic sites. Expression of the ρ2a subunit alone generated sufficient GABA-mediated currents (> 200 nA) with an EC50 of 0.6 ± 0.1 μM (Fig. 3a; Table 2). However, for unknown reasons, we failed to obtain GABA-evoked currents from oocytes injected with ρ1, ρ2b, ρ3a, or ρ3b subunit cRNAs.

**Table 1:** Expression patterns of GABA_A_ receptor subunit genes in zebrafish larvae at 5 dpf.

	Olfactory bulb	Forebrain	Midbrain	Hindbrain	Eye
α1		+++	+++	+++	+++
α2a		+	+		+
α3		+	+	+	
α4		+	++		+
α5		+	+		
α6a		+	+		
α6b	+	+	+		+
β1	+	+++	+++	+++	+++
β2	+	+++	+++	+++	+++
β3		++	++	+	++
β4		++	++	+	+
γ1		++	++		++
γ2	+	++	++	+	++
γ3		++	+	+	+
δ	++	++	+++	+	++
π/πa			+		+
ζ/πb		+	+	+	+
ρ1		+	+		++
ρ2a	++	+	++	+	+++
ρ2b		+	+		+
ρ3a		+	+		+
ρ3b	+	+	+	+	++

Note that +++, ++, and + indicate the intensity of staining.

**Table 2:** Summary of the gating properties of zebrafish GABA_A_ receptors.

	GABA_A_ receptor	EC50 (μM)	Hill coefficient
**α1**	β1	ND	ND
	β1γ2	ND	ND
	β2	ND	ND
	β2γ2	ND	ND
	β3	3.2 ± 0.2	1.6 ± 0.1
	β3γ2	126.9 ± 10.0	1.3 ± 0.1
	β4	3.6 ± 0.6	1.0 ± 0.1
	β4γ2	143.4 ± 12.5	1.2 ± 0.1
**α2a**	β1	ND	ND
	β1γ2	ND	ND
	β2	ND	ND
	β2γ2	ND	ND
	β3	4.6 ± 0.4	1.2 ± 0.1
	β3γ2	99.9 ± 14.1	1.3 ± 0.1
**α3**	β1	ND	ND
	β1γ2	ND	ND
	β2	ND	ND
	β2γ2	ND	ND
	β3	11.5 ± 2.1	1.1 ± 0.1
	β3γ2	239.6 ± 24.2	0.9 ± 0.0
**α4**	β1	ND	ND
	β1γ2	ND	ND
	β2	ND	ND
	β2γ2	ND	ND
	β3	ND	ND
	β3γ2	ND	ND
**α5**	β1	ND	ND
	β1γ2	ND	ND
	β2	ND	ND
	β2γ2	ND	ND
	β3	1.9 ± 0.2	1.4 ± 0.1
	β3γ2	66.6 ± 7.5	1.0 ± 0.1
**α6a**	β1	ND	ND
	β1γ2	ND	ND
	β2	ND	ND
	β2γ2	ND	ND
	β3	ND	ND
	β3γ2	ND	ND
**α6b**	β1	ND	ND
	β1γ2	ND	ND
	β2	ND	ND
	β2γ2	ND	ND
	β3	0.6 ± 0.2	1.6 ± 0.2
	β3γ2	7.0 ± 1.3	0.6 ± 0.0
**γ1**	α1β3	58.0 ± 4.0	1.5 ± 0.1
**γ3**	α1β3	9.0 ± 0.8	2.1 ± 0.3
**δ**	α1β3	ND	ND
**π/πa**	α1β3	2.8 ± 0.6	1.2 ± 0.2
**ζ/πb**	α1β3	ND	ND
ρ1		ND	ND
ρ2a		ND	ND
ρ2b		0.6 ± 0.1	0.9 ± 0.0
ρ3a		ND	ND
ρ3b		ND	ND

Values represent the mean ± SEM. ND: the maximum current smaller than 200 nA and, thus, not determined.

In mammals, heteropentameric GABA_A_ receptors are composed of two α and two β subunits, along with another one chosen from the γ, δ, ε, π or θ subunit^2,7^, with α1β2γ2 and α1β3γ2 being the two most prevalent subtypes in rat brain neurons^22^. Mammalian GABA_A_ receptors composed of only α and β subunits can also function as GABA-dependent Cl^−^ channels with lower EC50 values compared to those of αβγ GABA_A_ receptors in *Xenopus* oocytes^23^. To explore the electrophysiological properties of GABA_A_ receptor subtypes in zebrafish, we next recorded the GABA-mediated currents from oocytes injected with one α and one β cRNAs with or without one γ cRNA. Co-expression of the β3 subunit with the α1, α2a, α3, α5, or α6b subunit yielded GABA-evoked currents, whereas that with the α2b, α4, or α6a subunit did not (Fig. 3b). Similarly, co-expression of β3 and γ2 subunits with the α1, α2a, α3, α5, or α6b subunit generated GABA-mediated currents, while that with the α2b, α4, or α6a subunit did not (Fig. 3c). In all cases, co-expression of the γ2 subunit increased the EC50 value. Co-expression of the β4 with the α1 subunit in the absence or presence of the γ2 subunit also yielded GABA-evoked currents, the latter with a higher EC50 value (Fig. 3d, e). By contrast, for unknown reasons, co-expression of either β1 or β2 with any α subunit in the absence or presence of the γ2 subunit failed to elicit currents following exposure to GABA. We also tested all γ, δ, π/πa, and ζ/πb subunits to determine whether their co-expression changed the EC50 of α1β3 GABA_A_ receptors, implying the incorporation of these subunits into the functional heteropentameric channels. Co-expression of the α1 and β3 subunits with the γ1, γ2, or γ3 subunit generated GABA-evoked currents with higher EC50 values compared to those in α1β3 GABA_A_ receptors, while those with the π/πa subunit yielded currents with lower EC50 values (Fig. 3f). Interestingly, co-expression of α1 and β3 with either the δ or ζ/πb subunit eliminated GABA-dependent currents. These results showed that the electrophysiological sensitivity of zebrafish GABA_A_ receptors to GABA differed according to the subunit combination, providing the functional diversity of GABA_A_ receptor subtypes in zebrafish, as observed in mammals.

## Discussion

In this study, we investigated the phylogeny, expression, and electrophysiology of zebrafish GABA_A_ receptor subunits using in silico analysis, in situ hybridization, and in vitro current recording, respectively. These analyses revealed differences in the spatial expression and electrophysiological properties of GABA_A_ receptors in zebrafish and suggested the conservation of receptor characteristics with minor differences in vertebrates.

### Conservation of GABA receptor genes in vertebrates

Previous database searches have suggested the presence of 23 GABA_A_ receptor subunits/genes in zebrafish^12^. However, one of the annotated α2b/*gabra2b* exons was removed from the database as a result of standard genome annotation processing in NCBI (https://www.ncbi.nlm.nih.gov/protein/1040662547). In this study, we identified a new exon and corrected the α2b/*gabra2b* annotation. Our cloning of 23 cDNAs of zebrafish GABA_A_ receptor subunits confirmed that all of the exon–intron junctions were correct for the 22 previously suggested and 1 newly identified GABA_A_ receptor subunit. We noticed that the β4 subunit is found in zebrafish, amphibians, reptiles, and birds but not in mammals. Interestingly, the spatial expression pattern and electrophysiological properties of the β4 subunit were similar to those of the β3 subunit in zebrafish. Thus, β4 may serve as a reserve of β3 to form functionally indistinguishable GABA_A_ receptor subtypes. Our phylogenetic analyses also suggested that the zebrafish-specific ζ subunit is a paralog of the π subunit, presumably generated by gene duplication in teleosts^20^. Thus, our nomenclature of changing the π and ζ to πa and πb, respectively, is reasonable. We also noted that neither ε nor θ subunit is found in zebrafish, while the ε subunit is found only in mammals and birds and the θ subunit is found in mammals, birds, and reptiles.

A recent study proposed that a subfamily of ρ subunits is phylogenetically close to a subfamily comprising α, γ, and ε subunits^13^. However, our phylogenetic tree suggested that the ρ subfamily is instead close to a subfamily comprising the β, θ, δ, and π subunits, consistent with an old phylogenetic study^24^. This discrepancy could be caused by a difference in phylogenetic methods and, thus, will be solved in future development of phylogenetic methods.

### Spatial distributions of GABA_A_ receptor genes

A previous in situ hybridization study reported the expression patterns of GABA_A_ receptor subunit genes in zebrafish at 1, 2, and 4 dpf^13^. Here, we assayed the spatial expressions of α and the other subunit genes in zebrafish at 5 dpf. The nucleotide sequence identity of the GABA_A_ receptor coding regions was 41.6–75.0%, with the highest between ρ3a and ρ3b paralogs and the second highest (72.1%) between ρ2a and ρ2b paralogs (Supplementary Fig. 18). Although the expression patterns of ρ3a and ρ3b were almost identical, those of ρ2a and ρ2b were different at least in the olfactory bulb. Thus, we assume that each antisense probe presumably recognizes its specific target. The results of in situ hybridization showed that α1 was predominantly expressed in the larval brain at 5 dpf. Depletion of the α1 subunit in zebrafish affected spontaneous behavior at 5 dpf^14,15^. Thus, the α1 appears to be the major isoform in zebrafish, similar to that in mammals^22^. We also confirmed the overlapping expression patterns of the β3 and the γ2 with the α1 subunit in the larval forebrain, midbrain, and hindbrain, suggesting that the α1β3γ2 GABA_A_ receptor, which is the prevalent subtype in mammals, may be formed in zebrafish. However, our RNA labeling does not provide insights at the cellular level and thus, we cannot determine the actual co-expression.

### Electrophysiological properties of zebrafish GABA_A_ receptor subunits

Previous electrophysiological recordings of GABA-evoked currents from *Xenopus* oocytes expressing the human β3 subunit with the human α (α1–6) subunit enabled EC50 comparisons of different GABA_A_ receptor subtypes^23^. The order of the EC50 values for the human GABA_A_ receptor was α4β3 < α6β3 < α5β3<α1β3, α2β3, α3β3. Co-expression of the human γ2 subunit increased the EC50 values and mostly maintained the order: α6β3γ2, α5β3γ2 <  α4β3γ2, α1β3γ2, α2β3γ2, α3β3γ2. This finding is consistent with the fact that α1/2/3 subunit-containing phasic GABA_A_ receptors localize at synaptic sites where the GABA concentration increases to more than 1 mM during inhibitory transmission, while α4/5/6 subunit-containing tonic GABA_A_ receptors function at extrasynaptic sites where the GABA concentration is low (~ 0.5 µM)^2,7^. Similar oocyte recordings using zebrafish GABA_A_ receptor subunits in this study revealed an order of EC50 values of α6bβ3 < α5β3, α1β3, α2aβ3 < α3β3 in the absence of the γ2 subunit and α6bβ3γ2 < α5β3γ2, α2aβ3γ2, α1β3γ2 <  α3β3γ2 in the presence of the zebrafish γ2 subunit. Thus, the electrophysiological properties of each subunit appeared to be conserved among vertebrates, suggesting that α1/2/3- and α5/6b-containing GABA_A_ receptors are synaptic and extrasynaptic, respectively, in zebrafish. Co-expression of the γ2 subunit increased the EC50 values in both human and zebrafish, further supporting the notion that the electrophysiological properties of GABA_A_ receptor subunits are conserved in vertebrates.

Previous studies of oocyte electrophysiology have shown that β3 homopentameric GABA_A_ receptors produce small currents when exposed to 10 mM GABA and much larger currents when exposed to 100 µM etomidate^17,25^. We recapitulated that zebrafish β3 homopentameric GABA_A_ receptors elicited small currents upon exposure to 10 mM GABA and large currents upon exposure to 100 µM etomidate, with the latter showing an EC50 of 30.3 ± 5.7 µM (data not shown). Zebrafish homopentameric GABA_A_ receptors comprising the β1, β2 or β4 also produced small currents following exposure to 10 mM GABA, suggesting that either these β subunits can be expressed in *Xenopus* oocytes. However, heteropentameric zebrafish GABA_A_ receptors containing β3 or β4 yielded GABA-evoked currents, whereas those containing β1 or β2 did not. We were unable to determine why zebrafish β1 and β2 subunits failed to function in heteropentameric GABA_A_ receptors. A triple-knockout study of the GABA_A_ receptor β subunit in mice suggested that β3 plays an indispensable role in inhibitory synaptic transmission in mammals^26^. CRISPR-mediated disruption of the β3 gene in zebrafish increased spontaneous larval movements, also implying an essential physiological function of the β3 subunit in zebrafish^17^. Thus, β3 is presumably the primary β isoform not only in mammals but also in zebrafish.

The mammalian ρ subunit can form homopentameric GABA receptors, which were initially referred to as GABA_C_ receptors^27^ and eventually recategorized as GABA_A_ receptors^19^. Our electrophysiology also showed that zebrafish ρ2a subunit forms functional homopentameric GABA_A_ receptors. The expression of the ρ2a subunit was observed in zebrafish eyes, similar to the expression of the ρ2 subunit in mice^28^.

Although heteropentameric zebrafish α1β3 GABA_A_ receptors elicited GABA-evoked currents in *Xenopus* oocytes, the additional expression of either the δ or ζ/πb subunit eliminated the currents inconsistent with the findings in mammalian GABA_A_ receptor cases^23^. The zebrafish δ and ζ/πb subunits may suppress the formation of α1β3 heteropentameric channels. We also failed to record GABA-mediated currents from oocytes injected with α4, α6a, β1, β2, ρ1, ρ2b, ρ3a, or ρ3b cRNA inconsistent with the previous electrophysiology using mammalian orthologs of these subunits. The efficiency of zebrafish protein synthesis may differ among receptor subunits in *Xenopus* oocytes.

Taken together, our current study provides basic information on the expression and gating properties of zebrafish GABA_A_ receptors. Since recent development in CRISPR/Cas9 technology have enabled easy and multiple targeted gene disruption, future studies of GABA_A_ receptor knockout in zebrafish will clarify the physiologically relevant function of each GABA_A_ receptor subunit and unveil the significance of GABA_A_ receptor diversity.

## Materials and methods

### Animals

Zebrafish (*Danio rerio*) were reared and maintained in 1.7 L tanks in a recirculating Meito System (Meito System) at 28.5 °C under a 14 h light and 10 h dark photoperiod according to the standard protocol^29^. Larvae were fed paramecia and Gemma Micro ZF 75 (Funakoshi) twice daily from 5 to 30 dpf. Juvenile fish were fed brine shrimp (Tokai Guppy) and Gemma Micro ZF 75 twice daily from 30 to 90 dpf. Adult fish were fed brine shrimp and Otohime B2 (Marubeni Nissin Feed) twice daily after 90 dpf. Zebrafish AB line was purchased from the Zebrafish International Resource Center (https://zebrafish.org/home/guide.php) and used for line maintenance.

### Phylogenetic analysis

Amino acid sequences of human and mouse GABA_A_ receptor subunit proteins were obtained from the NCBI database. The accession numbers are as follows. Human GABA_A_ receptor subunit: α1: NP_000797; α2: NP_000798; α3: NP_000799; α4: NP_000800; α5: NP_000801; α6: NP_000802; β1: NP_000803; β2: NP_000804; β3: NP_000805; γ1: NP_775807; γ2: NP_944494; γ3: NP_150092; ε: NP_004952; δ: NP_000806; π: NP_055026; θ: NP_061028; ρ1: NP_002033; ρ2: NP_002034; and ρ3: NP_001099050. Mouse GABA_A_ receptor subunit: α1: NP_001345964; α2: NP_032092; α3: NP_001344743; α4: NP_034381; α5: NP_001349090; α6: NP_001093111; β1: NP_032095; β2: NP_001349575; β3: NP_032097; γ1: NP_034382; γ2: NP_032099; γ3: NP_032100; ε: NP_059065; δ: NP_032098; π: NP_666129; θ: NP_065234; ρ1: NP_032101; ρ2: NP_032102; and ρ3: NP_001074659. Zebrafish GABA_A_ receptor subunit: α1: NP_001070794; α2a: XP_009305482; α2b: LC596832; α3: XP_021324930; α4: NP_001017822; α5: XP_005166139; α6a: NP_957025; α6b: XP_002667403; β1: XP_002664179; β2: XP_016092780; β3: XP_005166138; β4: XP_017208500; γ1: XP_009305483; γ2: NP_001243179; γ3: XP_009300843; δ: XP_700099; π/πa: XP_002664479; ζ/πb: NP_001108214; ρ1: NP_001020724; ρ2a: XP_017207163; ρ2b: XP_697486; ρ3a: XP_009295726; and ρ3b: NP_001122232. To create a phylogenetic tree, we used the Interactive Tree of Life (iTOL) online tool v5 (https://itol.embl.de).

### Cloning of GABA_A_ receptor subunits

Total RNA was extracted from mixtures of 1, 2, and 3 dpf zebrafish embryos and 4 and 5 dpf larvae using Sepasol RNA II Super (Nacalai Tesque) as described previously^30^. Oligo(dT)18 Primer (Thermo Fisher Scientific), SuperScript IV Reverse Transcriptase (Thermo Fisher Scientific), and Phusion DNA polymerase (New England Biolabs) were used for RT-PCR as described previously^31^. The primer sequences are listed in Supplementary Table 2. The following program was used for amplification: 94 °C for 2 min; 94 °C for 10 s, 63 °C for 20 s, 72 °C for 30 s, 35 cycles of 72 °C for 1 min, and 4 °C forever. The PCR products were cloned into the pCS2 + expression vector as described previously^32^.

### Whole-mount in situ hybridization

In situ hybridization of whole-mount zebrafish embryos with a digoxigenin-labeled antisense RNA probe was performed as described previously^33^. Digoxigenin-labeled probes covering the complete coding sequences were used (α1: 1377 bp; α2a: 1353 bp; α2b: 1353 bp; α3: 1425 bp; α4: 1671 bp; α5: 1359 bp; α6a: 1332 bp; α6b: 1305 bp; β1: 1452 bp; β2: 1416 bp; β3: 1494 bp; β4: 1446 bp; γ1: 1362 bp; γ2: 1428 bp; γ3: 1392 bp; δ: 1377 bp; π/πa: 1335 bp; ζ/πb: 1341 bp; ρ1: 1398 bp; ρ2a: 1422 bp; ρ2b: 1392 bp; ρ3a: 1416 bp; and ρ3b: 1413 bp).

### In vitro synthesis of capped cRNAs

Capped zebrafish GABA receptor mRNAs were synthesized from pCS2 + based plasmids using the mMessage mMachine SP6 Transcription Kit (Thermo Fisher Scientific) as described previously^34^.

### Electrophysiology

Electrophysiology was performed as described previously^35^. In brief, oocytes were injected with five femtomoles of cRNAs using a Nanoject II (Drummond Scientific) and incubated in Barth’s solution (88 mM NaCl, 1 KCl, 2.4 mM NaHCO_3_, 0.33 mM Ca(NO_3_)_2_, 0.41 mM CaCl_2_, 0.82 mM MgSO_4_, and 10 mM HEPES at pH 7.5 with NaOH supplemented with gentamicin at 50 µL/mL and penicillin/streptomycin at 100 units/mL) at 17 °C for 24–72 h before recording. Oocyte recording solution (90 mM NaCl, 1 mM KCl, 2 mM CaCl_2_, 1 mM MgCl_2_, and 10 mM HEPES at pH 7.5 with NaOH) and GABA solutions of different concentrations were applied to oocytes using a BPS-8 solution switcher (ALA Scientific). The borosilicate electrodes had resistances of ~ 0.5 MΩ when filled with 3 M KCl. Two-electrode voltage-clamp recordings were made from oocytes held at -50 mV using pClamp 10.2 to control GeneClamp 500B amplifier via Digidata 1440A digitizer (Molecular Devices). Signals were low-pass filtered at 10 Hz and sampled at 100 Hz. The recordings were analyzed using Clampfit 10.7 (Axon Instruments) and SigmaPlot 11.0 (Systat Software). The sample numbers are indicated in the figures. The EC50s and Hill coefficients were calculated using the sigmoid standard curve as below. *x*: GABA concentration (EC50). y: normalized current.

$$\mathrm{y }=\mathrm{ min}+\frac{(\mathrm{max} - \mathrm{min})}{1+{(x/EC50)}^{-Hillslope}}$$

### Statistics

Quantitative data are presented as means ± SEM. All error bars in the graphs represent the SEM values. Statistical significance was determined by pairwise analysis of variance.

### Ethics statement

This study was approved by the Animal Care and Use Committee of Aoyama Gakuin University (A9/2020) and carried out according to the Aoyama Gakuin University Animal Care and Use Guidelines and the Animal Research of in vivo Experiments (ARRIVE) guidelines.

## Supplementary Information

Supplementary Information.

## References

[1] Smart TG, Stephenson FA (2019). A half century of gamma-aminobutyric acid. Brain Neurosci. Adv..

[2] Mortensen M, Patel B, Smart TG (2011). GABA potency at GABA(A) receptors found in synaptic and extrasynaptic zones. Front. Cell Neurosci..

[3] Mozrzymas JW, Zarnowska ED, Pytel M, Mercik K (2003). Modulation of GABA(A) receptors by hydrogen ions reveals synaptic GABA transient and a crucial role of the desensitization process. J. Neurosci..

[4] Lee S (2010). Channel-mediated tonic GABA release from glia. Science.

[5] Santhakumar V, Hanchar HJ, Wallner M, Olsen RW, Otis TS (2006). Contributions of the GABAA receptor alpha6 subunit to phasic and tonic inhibition revealed by a naturally occurring polymorphism in the alpha6 gene. J. Neurosci..

[6] Farrar SJ, Whiting PJ, Bonnert TP, McKernan RM (1999). Stoichiometry of a ligand-gated ion channel determined by fluorescence energy transfer. J. Biol. Chem..

[7] Mohler H (2006). GABA(A) receptor diversity and pharmacology. Cell Tissue Res..

[8] Olsen RW (2018). GABAA receptor: positive and negative allosteric modulators. Neuropharmacology.

[9] Naffaa MM, Hung S, Chebib M, Johnston GAR, Hanrahan JR (2017). GABA-rho receptors: distinctive functions and molecular pharmacology. Br. J. Pharmacol..

[10] Slany A, Zezula J, Tretter V, Sieghart W (1995). Rat beta 3 subunits expressed in human embryonic kidney 293 cells form high affinity [35S]t-butylbicyclophosphorothionate binding sites modulated by several allosteric ligands of gamma-aminobutyric acid type A receptors. Mol. Pharmacol..

[11] Grunwald DJ, Eisen JS (2002). Headwaters of the zebrafish—emergence of a new model vertebrate. Nat. Rev. Genet..

[12] Cocco A (2017). Characterization of the gamma-aminobutyric acid signaling system in the zebrafish (Danio rerio Hamilton) central nervous system by reverse transcription-quantitative polymerase chain reaction. Neuroscience.

[13] Monesson-Olson B (2018). Expression of the eight GABAA receptor alpha subunits in the developing zebrafish central nervous system. PLoS ONE.

[14] Reyes-Nava NG, Yu HC, Coughlin CR, Shaikh TH, Quintana AM (2020). Abnormal expression of GABAA receptor subunits and hypomotility upon loss of gabra1 in zebrafish. Biol Open.

[15] Samarut E (2018). gamma-Aminobutyric acid receptor alpha 1 subunit loss of function causes genetic generalized epilepsy by impairing inhibitory network neurodevelopment. Epilepsia.

[16] Gonzalez-Nunez V (2015). Role of gabra2, GABAA receptor alpha-2 subunit CNS development. Biochem. Biophys. Rep..

[17] Yang X (2019). Drug-selective anesthetic insensitivity of zebrafish lacking gamma-aminobutyric acid type A receptor beta3 subunits. Anesthesiology.

[18] Roy B, Ali DW (2014). Multiple types of GABAA responses identified from zebrafish Mauthner cells. NeuroReport.

[19] Alexander SP (2017). The concise guide to pharmacology 2017/18: ligand-gated ion channels. Br. J. Pharmacol..

[20] Amores A (1998). Zebrafish hox clusters and vertebrate genome evolution. Science.

[21] Baraban SC, Taylor MR, Castro PA, Baier H (2005). Pentylenetetrazole induced changes in zebrafish behavior, neural activity and c-fos expression. Neuroscience.

[22] Fritschy JM, Mohler H (1995). GABAA-receptor heterogeneity in the adult rat brain: differential regional and cellular distribution of seven major subunits. J. Comp. Neurol..

[23] Karim N (2013). Potency of GABA at human recombinant GABA(A) receptors expressed in Xenopus oocytes: a mini review. Amino Acids.

[24] Sigel E, Steinmann ME (2012). Structure, function, and modulation of GABA(A) receptors. J. Biol. Chem..

[25] Cestari IN, Uchida I, Li L, Burt D, Yang J (1996). The agonistic action of pentobarbital on GABAA beta-subunit homomeric receptors. NeuroReport.

[26] Nguyen QA, Nicoll RA (2018). The GABAA receptor beta subunit is required for inhibitory transmission. Neuron.

[27] Cutting GR (1991). Cloning of the gamma-aminobutyric acid (GABA) rho 1 cDNA: a GABA receptor subunit highly expressed in the retina. Proc. Natl. Acad. Sci. USA.

[28] Cutting GR (1992). Identification of a putative gamma-aminobutyric acid (GABA) receptor subunit rho2 cDNA and colocalization of the genes encoding rho2 (GABRR2) and rho1 (GABRR1) to human chromosome 6q14-q21 and mouse chromosome 4. Genomics.

[29] Westerfield M (2007). The Zebrafish Book: A Guide for the Laboratory Use of Zebrafish (*Danio rerio*).

[30] Hirata H, Ogino K, Yamada K, Leacock S, Harvey RJ (2013). Defective escape behavior in DEAH-box RNA helicase mutants improved by restoring glycine receptor expression. J. Neurosci..

[31] Hirata H (2007). Zebrafish relatively relaxed mutants have a ryanodine receptor defect, show slow swimming and provide a model of multi-minicore disease. Development.

[32] Ogino K (2015). RING finger protein 121 facilitates the degradation and membrane localization of voltage-gated sodium channels. Proc. Natl. Acad. Sci. USA.

[33] Hirata H (2004). accordion, a zebrafish behavioral mutant, has a muscle relaxation defect due to a mutation in the ATPase Ca2+ pump SERCA1. Development.

[34] Hirata H (2005). Zebrafish bandoneon mutants display behavioral defects due to a mutation in the glycine receptor beta-subunit. Proc. Natl. Acad. Sci. USA.

[35] Ito D, Kawazoe Y, Sato A, Uesugi M, Hirata H (2020). Identification of the hypertension drug niflumic acid as a glycine receptor inhibitor. Sci. Rep..

