# Supplementary material for: Characterization of zebrafish GABAA receptor subunits
Source: Sci Rep. 2021 Mar 18;11:6242. doi: 10.1038/s41598-021-84646-3 (PMC7973766; doi:10.1038/s41598-021-84646-3)
Supplement: Supplementary file 1 — Supplementary Information. [file 41598_2021_84646_MOESM1_ESM.pdf]

Characterization of zebrafish $GABA_A$ receptor subunits

Kenichiro Sadamitsu, Leona Shigemitsu, Marina Suzuki, Daishi Ito, Makoto Kashima, and Hiromi Hirata[*]

Department of Chemistry and Biological Science, College of Science and Engineering, Aoyama Gakuin University, Sagamihara 252-5258, Japan

[*]**Correspondence:** Hiromi Hirata hihirata@chem.aoyama.ac.jp

Number of Supplementary Tables: 2
Number of Supplementary Figures: 18

Supplementary Table 1. Amino acid identities of human and zebrafish $GABA_A$ receptor proteins.

Human	Zebrafish	Signal peptide	Transmembrane	The other regions
α1	α1	30%	97%	87%
α2	α2a	11%	98%	82%
	α2b	11%	98%	84%
α3	α3	0%	97%	55%
α4	α4	46%	99%	62%
α5	α5	23%	98%	77%
α6	α6a	26%	82%	58%
	α6b	21%	91%	69%
β1	β1	8%	92%	82%
β2	β2	28%	92%	86%
β3	β3	60%	96%	77%
	β4	40%	92%	71%
γ1	γ1	26%	92%	76%
γ2	γ2	26%	95%	88%
γ3	γ3	41%	98%	77%
δ	δ	31%	91%	73%
π	π/πa	25%	79%	51%
ζ	ζ/πb	6%	80%	51%
ρ1	ρ1	0%	95%	71%
ρ2	ρ2a	30%	90%	81%
	ρ2b	5%	92%	71%
ρ3	ρ3a	21%	81%	65%
	ρ3b	17%	79%	64%

Supplementary Table 2. Oligonucleotide primers for the zebrafish $GABA_A$ receptor subunit cloning used in this study.

Primer		Sequence	Restriction enzyme
α1	forward	CTCTGAATTCGCCGCCACCATGATGTG GGGTGGAAGAGGAGCA	EcoRI
	reverse	GAGATCTAGATTAGTGTGGTTGTAGGT TCATACCCTG	XbaI
α2a	forward	CTCTGAATTCGCCGCCACCATGATTCT GCGACGGGGTCATTCTCA	EcoRI
	reverse	GAGACTCGAGTCACGGGGAGGGTACC ATGTTC	XhoI
α2b	forward	GAGAGAGAATCGATGCCGCCACCATG AGGGAGAGGTTCGCGGTTCATCTTC	ClaI
	reverse	GTGTGTGTCTCGAGTTAATGGGACGG CACCATGTCTTTAATC	XhoI
α3	forward	CTCTGAATTCGCCGCCACCATGGCTGG CGTTTTCCACAAGC	EcoRI
	reverse	GAGACTCGAGTCATTTAGGTGGGCTG GACCGC	XhoI
α4	forward	ATGGTTTCTGCCAAGAAGGAGATGGT GACTGC	EcoRI
	reverse	TTAGGCTCCCTTGGCCTCCATGGTGTC TTTG	XhoI
α5	forward	CTCTGAATTCGCCGCCACCATGGGAT ATGGACCACACTGCAGC	EcoRI
	reverse	GAGACTCGAGCTAAACGGCTCCTTTA ATCACTGG	XhoI
α6a	forward	CTCTGAATTCGCCGCCACCATGGCTCT ACTTTTGGCCTTTTTA	EcoRI
	reverse	GAGACTCGAGTCACTCGATATCCCTG AGCTTTTCC	XhoI
α6b	forward	CTCTGAATTCGCCGCCACCATGGCTTG GGTACGCACATGCC	EcoRI
	reverse	GAGACTCGAGTCACACTTCCCTGGATT CCATTGTGTC	XhoI
β1	forward	CTCTGAATTCGCCGCCACCATGATGA GAGGGATGCGGATCGCC	EcoRI
	reverse	GAGATCTAGACTAGTGGACATAATAA AGCCAGTAAAC	XbaI
β2	forward	CTCTGAATTCGCCGCCACCATGGAGA GTATCGGAAAAACCCCAC	EcoRI
	reverse	GAGACTCGAGTTAATGAACGTAGTAA AGCCAGTAAAC	XhoI
β3	forward	CTCTGAATTCGCCGCCACCATGTTTGG AATACAGAAACGAAGG	EcoRI
	reverse	GAGACTCGAGCTAATTCACATAATAC AGCCAATAGATC	XhoI

β4	forward	CTCTGAATTCGCCGCCACCATGTTGGG TCCTCAAGAAGACAAATT	EcoRI
	reverse	GAGATCTAGACTAATTCACATAATAC AACCAATAGATAAGG	XbaI
γ1	forward	CTCTGAATTCGCCGCCACCATGGTTCA TCTGCATCCCCGCCGAAG	EcoRI
	reverse	GAGACTCGAGTTAAAGATACAGATAT CCAATCCAGTAGAC	XhoI
γ2	forward	CTCTGAATTCGCCGCCACCATGGTGAT GATGGCTTCTCTTCATTTT	EcoRI
	reverse	GAGATCTAGATTAAAGATAGAGATAG GAAAACCAGTAAACTACG	XbaI
γ3	forward	CTCTGAATTCGCCGCCACCATGACCAC CAAATTGTTCCTCTACTTC	EcoRI
	reverse	GAGACTCGAGCTAAAGATACAAGTAG CCTACCCAG	XhoI
δ	forward	CTCTGAATTCGCCGCCACCATGGATGC GCTGAGTTTGACGGTG	EcoRI
	reverse	GAGACTCGAGCTACATGGTGTACGCC ACCCAGTAG	XhoI
π/πa	forward	CTCTGAATTCGCCGCCACCATGATCTG CATCTCAGATCTCCACAGC	EcoRI
	reverse	GAGACTCGAGTTAGAATAAAAGGTAG TATAGCCAGTAG	XhoI
ζ/πb	forward	CTCTGAATTCGCCGCCACCATGCATGT GTGTAGCTTCGTGGGC	EcoRI
	reverse	GAGACTCGAGTTAAAAGTACAAGTAG TAGGTCCAGTAGTGG	XhoI
ρ1	forward	CTCTGAATTCGCCGCCACCATGCACGC GGATGTCTGCTTTCTGC	EcoRI
	reverse	GAGACTCGAGTCACTGTGAGTAGATG GACCAGTATATG	XhoI
ρ2a	forward	CTCTGAATTCGCCGCCACCATGCCTTA TGCGAGACAGCTCC	EcoRI
	reverse	GAGACTCGAGTCAGCAGTACACCGAC CAGTAAATAAGG	XhoI
ρ2b	forward	CTCTGAATTCGCCGCCACCATGACTGT CCTTTTTCTGAAAGCG	EcoRI
	reverse	GAGACTCGAGTCAGAGGTAGACAGAC CAGTAGATGAG	XhoI
ρ3a	forward	CTCTGAATTCGCCGCCACCATGAAGCT GGTCCTGCTGACGCTCAGG	XhoI
	reverse	GAGACTCGAGTCAAGAATAGATGCTC CAGTAGATGAGATTGAAGAGAACG	XhoI
ρ3b	forward	CTCTGAATTCGCCGCCACCATGAAGCT GGTCCTGCTGACGCTC	EcoRI
	reverse	GAGACTCGAGTCAAGAATAGATGCTC CAGTAGATGAGA	XhoI

Supplemental Figure 1. Alignments of the GABA$_A$ receptor α1 subunits.
Amino acid alignments of human, mouse, and zebrafish GABA$_A$ receptor α1 subunits. All of the GABA$_A$ receptor subunits have an N-terminal signal peptide and four transmembrane domains (M1–M4). The transmembrane domains are highly conserved among vertebrates.

Supplemental Figure 2. Alignments of GABA$_A$ receptor α2 subunits.
Amino acid alignments of human, mouse, and zebrafish GABA$_A$ receptor α2 subunits. All of the GABA$_A$ receptor subunits have an N-terminal signal peptide and four transmembrane domains (M1–M4). The transmembrane domains are highly conserved among vertebrates.

Supplemental Figure 3. Alignments of GABA$_A$ receptor α3 subunits.
Amino acid alignments of human, mouse, and zebrafish GABA$_A$ receptor α3 subunits. All of the GABA$_A$ receptor subunits have an N-terminal signal peptide and four transmembrane domains (M1–M4). The transmembrane domains are highly conserved among vertebrates.

Supplemental Figure 4. Alignments of GABA$_A$ receptor α4 subunits.
Amino acid alignments of human, mouse, and zebrafish GABA$_A$ receptor α4 subunits. All of the GABA$_A$ receptor subunits have an N-terminal signal peptide and four transmembrane domains (M1–M4). The transmembrane domains are highly conserved among vertebrates.

Supplemental Figure 5. Alignments of GABA$_A$ receptor α5 subunits.
Amino acid alignments of human, mouse, and zebrafish GABA$_A$ receptor α5 subunits. All of the GABA$_A$ receptor subunits have an N-terminal signal peptide and four transmembrane domains (M1–M4). The transmembrane domains are highly conserved among vertebrates.

Supplemental Figure 6. Alignments of GABA$_A$ receptor α1 subunits.
Amino acid alignments of human, mouse, and zebrafish GABA$_A$ receptor α6 subunits. All of the GABA$_A$ receptor subunits have an N-terminal signal peptide and four transmembrane domains (M1–M4). The transmembrane domains are highly conserved among vertebrates.

Supplemental Figure 7. Alignments of $GABA_A$ receptor β1 subunits.

Amino acid alignments of human, mouse, and zebrafish $GABA_A$ receptor β1 subunits. All of the $GABA_A$ receptor subunits have an N-terminal signal peptide and four transmembrane domains (M1–M4). The transmembrane domains are highly conserved among vertebrates.

Supplemental Figure 8. Alignments of $GABA_A$ receptor β2 subunits.

Amino acid alignments of human, mouse, and zebrafish $GABA_A$ receptor β2 subunits. All of the $GABA_A$ receptor subunits have an N-terminal signal peptide and four transmembrane domains (M1–M4). The transmembrane domains are highly conserved among vertebrates.

Supplemental Figure 9. Alignments of $GABA_A$ receptor β3 subunits.

Amino acid alignments of human, mouse, and zebrafish $GABA_A$ receptor β3 and β4 subunits. All of the $GABA_A$ receptor subunits have an N-terminal signal peptide and four transmembrane domains (M1–M4). The transmembrane domains are highly conserved among vertebrates.

Supplemental Figure 10. Alignments of $GABA_A$ receptor γ1 subunits.

Amino acid alignments of human, mouse, and zebrafish $GABA_A$ receptor γ1 subunits. All of the $GABA_A$ receptor subunits have an N-terminal signal peptide and four transmembrane domains (M1–M4). The transmembrane domains are highly conserved among vertebrates.

Supplemental Figure 11. Alignments of $GABA_A$ receptor γ2 subunits.

Amino acid alignments of human, mouse, and zebrafish $GABA_A$ receptor γ2 subunits. All of the $GABA_A$ receptor subunits have an N-terminal signal peptide and four transmembrane domains (M1–M4). The transmembrane domains are highly conserved among vertebrates.

Supplemental Figure 12. Alignments of $GABA_A$ receptor γ3 subunits.

Amino acid alignments of human, mouse, and zebrafish $GABA_A$ receptor γ3 subunits. All of the $GABA_A$ receptor subunits have an N-terminal signal peptide and four transmembrane domains (M1–M4). The transmembrane domains are highly conserved among vertebrates.

Supplemental Figure 13. Alignments of $GABA_A$ receptor δ subunits.

Amino acid alignments of human, mouse, and zebrafish $GABA_A$ receptor δ subunits. All of the $GABA_A$ receptor subunits have an N-terminal signal peptide and four transmembrane domains (M1–M4). The transmembrane domains are highly conserved among vertebrates.

Supplemental Figure 14. Alignments of $GABA_A$ receptor π subunits.

Amino acid alignments of human, mouse, and zebrafish $GABA_A$ receptor π subunits. All of the $GABA_A$ receptor subunits have an N-terminal signal peptide and four transmembrane domains (M1–M4). The transmembrane domains are highly conserved among vertebrates.

Supplemental Figure 15. Alignments of $GABA_A$ receptor ρ1 subunits.

Amino acid alignments of human, mouse, and zebrafish $GABA_A$ receptor ρ1 subunits. All of the $GABA_A$ receptor subunits have an N-terminal signal peptide and four transmembrane domains (M1–M4). The transmembrane domains are highly conserved among vertebrates.

Supplemental Figure 16. Alignments of $GABA_A$ receptor ρ2 subunits.

Amino acid alignments of human, mouse, and zebrafish $GABA_A$ receptor ρ2 subunits. All of the $GABA_A$ receptor subunits have an N-terminal signal peptide and four transmembrane domains (M1–M4). The transmembrane domains are highly conserved among vertebrates.

Supplemental Figure 17. Alignments of $GABA_A$ receptor ρ3 subunits.

Amino acid alignments of human, mouse, and zebrafish $GABA_A$ receptor ρ3 subunits. All of the $GABA_A$ receptor subunits have an N-terminal signal peptide and four transmembrane domains (M1–M4). The transmembrane domains are highly conserved among vertebrates.

Supplemental Figure 18. Nucleotide identities of the coding sequences of zebrafish $GABA_A$ receptors.

The percent nucleotide identities of the coding sequences of zebrafish $GABA_A$ receptors are indicated in the matrix. The highest and lowest values are 75.0% (ρ3a and ρ3b) and 41.6% (α6a and β2), respectively.

GABA$_A$ receptor subunit α1

SP

α1	Human	1	MRKSPGLSDCLWAWI--LLLSTLTGRSYGQPSLQDELKDNTTVFTRILDRLLDGYDNRLR	58
α1	Mouse	1	MKKSRGLSDYLWAWT--LILSTLSGRSYGQPS-QDELKDNTTVFTRILDRLLDGYDNRLR	57
α1	Zebrafish	1	MMWGGRGAAWLWIWACLLVTNALAGKSSSQNA--DEQKDNTTVFTRILDSLLDGYDNRLR	58

α1	Human	59	PGLGERVTEVKTDIFVTSFGPVSDHDMEYTIDVFFRQSWKDERLKFKGPMTVLRLNNLMA	118
α1	Mouse	58	PGLGERVTEVKTDIFVTSFGPVSDHDMEYTIDVFFRQSWKDERLKFKGPMTVLRLNNLMA	117
α1	Zebrafish	59	PGLGERVTEVKTDIFVTSIGPVSDHDMEYTIDVFFRQSWKDERLKFKGPMAVLRLNNLMA	118

α1	Human	119	SKIWTPDTFFHNGKKSVAHNMTMPNKLLRITEDGTLLYTMRLTVRAECPMHLEDFPMDAH	178
α1	Mouse	118	SKIWTPDTFFHNGKKSVAHNMTMPNKLLRITEDGTLLYTMRLTVRAECPMHLEDFPMDAH	177
α1	Zebrafish	119	SKIWTPDTFFHNGKKSVAHNMTMPNKLLRITEEGTLLYTMRLTVRAECPMHLEDFPMDAH	178

α1	Human	179	ACPLKFGSYAYTRAEVVYEWTREPARSVVVAEDGSRLNQYDLLGQTVDSGIVQSSTGEYV	238
α1	Mouse	178	ACPLKFGSYAYTRAEVVYEWTREPARSVVVAEDGSRLNQYDLLGQTVDSGIVQSSTGEYV	237
α1	Zebrafish	179	ACPLKFGSYAYTRAEVVYVWTRGAAQSVVVADDGSRLNQYDLMGQSVDSGVVQSSTGEYV	238

M1 M2

α1	Human	239	VMTTHFHLKRKIGYFVIQTYLPCIMTVILSQVSFWLNRESVPARTVFGVTTVLTMTTLSI	298
α1	Mouse	238	VMTTHFHLKRKIGYFVIQTYLPCIMTVILSQVSFWLNRESVPARTVFGVTTVLTMTTLSI	297
α1	Zebrafish	239	VMTTHFHLKRKIGYFVIQTYLPCIMTVILSQVSFWLNRESVPARTVFGVTTVLTMTTLSI	298

M3

α1	Human	299	SARNSLPKVAYATAMDWFIAVCYAFVFSALIEFATVNYFTKRGYAWDGKSVVPEKPKKVK	358
α1	Mouse	298	SARNSLPKVAYATAMDWFIAVCYAFVFSALIEFATVNYFTKRGYAWDGKSVVPEKPKKVK	357
α1	Zebrafish	299	SARNSLPKVAYATAMDWFIAVCYAFVFSALIEFATVNYFTKRGYAWDGKSVVPEKQKKKK	358

α1	Human	359	DPLIKKNNTY-APTATSYTPNLARGDPGLATIAKSATIEPKEVK--PETKPPEPKKTFNS	415
α1	Mouse	358	DPLIKKNNTY-APTATSYTPNLARGDPGLATIAKSATIEPKEVK--PETKPPEPKKTFNS	414
α1	Zebrafish	359	ESLLKKNNTYTAATATAFAPNIAR-DPGLATIAKSAPPPPTEPKEEPKPKPPEAKKTFNS	417

M4

α1	Human	416	VSKIDRLSRIAFPLLFGIFNLVYWATYLNREPQLKAPTPHQ-	456
α1	Mouse	415	VSKIDRLSRIAFPLLFGIFNLVYWATYLNREPQLKAPTPHQ-	455
α1	Zebrafish	418	VSKIDRIARIAFPLLFGTFNLVYWATYLNKKPKLQGMNLQPH	459

Sadamitsu et al. Supplementary Figure 1

GABA$_A$ receptor subunit α2

```
                             SP
α2   Human       1   ----MKTKLNIYNMQFLLFVFLVWDPARLVLANIQEDEAKNNITIFTRILDRLLDGYDNR   56
α2   Mouse       1   ----MKTKLSTCNVWSLLLVLLVWDPVRLVLANIQEDEAKNNITIFTRILDRLLDGYDNR   56
α2a  Zebrafish   1   MILRRGHSHGFLIHL--FLLVL-WRACSEA-EVSGSDAFKNNITLFTRILDRLLDGYDNR   56
α2b  Zebrafish   1   ------MRERFAVHLLVFALTLFWHTRSVS-ADATTDAFKNNITLFTRILDRLLDGYDNR   53

α2   Human      57   LRPGLGDSITEVFTNIYVTSFGPVSDTDMEYTIDVFFRQKWKDERLKFKGPMNILRLNNL  116
α2   Mouse      57   LRPGLGDSITEVFTNIYVTSFGPVSDTDMEYTIDVFFRQKWKDERLKFKGPMNILRLNNL  116
α2a  Zebrafish  57   LRPGLGDRVTEVKTDIYVTSFGPVSDTDMEYTVDVFFRQRWTDERLKFHGPMNILRLNNL  116
α2b  Zebrafish  54   LRPGLGDRVTTVKTDIYVTSFGPVSDTDMEYTIDVFFRQSWKDERLKFEGPMNILRLNNL  113

α2   Human     117   MASKIWTPDTFFHNGKKSVAHNMTMPNKLLRIQDDGTLLYTMRLTVQAECPMHLEDFPMD  176
α2   Mouse     117   MASKIWTPDTFFHNGKKSVAHNMTMPNKLLRIQDDGTLLYTMRLTVQAECPMHLEDFPMD  176
α2a  Zebrafish 117   MASKIWTPDTFFHNGKKSVAHNMTMPNKLLRIMENGTLLYTMRLTVQAECPMHLEDFPMD  176
α2b  Zebrafish 114   MASKIWTPDTFFHNGKKSVAHNMTMPNKLLRIQDDGTLLYTMRLTVHAECPMHLEDFPMD  173

α2   Human     177   AHSCPLKFGSYAYTTSEVTYIWTYNASDSVQVAPDGSRLNQYDLLGQSIGKETIKSSTGE  236
α2   Mouse     177   AHSCPLKFGSYAYTTSEVTYIWTYNASDSVQVAPDGSRLNQYDLLGQSIGKETIKSSTGE  236
α2a  Zebrafish 177   FHSCPLKFGSYAYTLTEVTYVWTRNATLSVEVAPDGSRLNQYDLMGQTVGKETIKSSTGE  236
α2b  Zebrafish 174   FHSCPLKFGSYAYTTTEVTYTWTKNASNSVVVEEESSRLNQYDLLGQTVGNETIRSSTGE  233
                                             M1                      M2
α2   Human     237   YTVMTAHFHLKRKIGYFVIQTYLPCIMTVILSQVSFWLNRESVPARTVFGVTTVLTMTTL  296
α2   Mouse     237   YTVMTAHFHLKRKIGYFVIQTYLPCIMTVILSQVSFWLNRESVPARTVFGVTTVLTMTTL  296
α2a  Zebrafish 237   YTVMTAHFHLKRKIGYFVIQTYLPCIMTVILSQVSFWLNRESVPARTVFGVTTVLTMTTL  296
α2b  Zebrafish 234   YTVMTAHFHLKRKIGYFVIQTYLPCIMTVILSQVSFWLNRESVPARTVFGVTTVLTMTTL  293
                                              M3
α2   Human     297   SISARNSLPKVAYATAMDWFIAVCYAFVFSALIEFATVNYFTKRGWAWDGKSVVNDKKKE  356
α2   Mouse     297   SISARNSLPKVAYATAMDWFIAVCYAFVFSALIEFATVNYFTKRGWAWDGKSVVNDKKKE  356
α2a  Zebrafish 297   SISARNSLPKVAYATAMDWFIAVCYAFVFSALIEFATVNYFTKRGWAWDGKSIVNDKKKE  356
α2b  Zebrafish 294   SISARNSLPKVAYATAMDWFIAVCYAFVFSALIEFATVNYFTKRGWAWDGKSVVNDKKKE  353

α2   Human     357   KASVMIQNNAYAVAVANYAPNLSKDPVLSTISKSATTPEPNKKPENKPAEAKKTFNSVSK  416
α2   Mouse     357   KGSVMIQNNAYAVAVANYAPNLSKDPVLSTISKSATTPEPNKKPENKPAEAKKTFNSVSK  416
α2a  Zebrafish 357   -TSVMKKNNAYAVAVANYAPHIAKDSALPTVSKSATPEPSKAQPVAK--ETKKTFNSVSK  413
α2b  Zebrafish 354   KASVIKKNNAYAVAVANYAPNITKDTVLPTIAKGSVAEQGKTKVDGKPAEAKKTFNSVSK  413
                             M4
α2   Human     417   IDRMSRIVFPVLFGTFNLVYWATYLNREPVLGVSP---                        451
α2   Mouse     417   IDRMSRIVFPVLFGTFNLVYWATYLNREPVLGVSP---                        451
α2a  Zebrafish 414   IDRMSRIIFPVLFGSFNLVYWATYLNREPVIKNMVPSP                        451
α2b  Zebrafish 414   IDRMSRIMFPVLFGSFNLVYWATYLNREPVIKDMVPSH                        451
```

Sadamitsu et al. Supplementary Figure 2

GABA$_A$ receptor subunit α3

```
                              SP
α3  Human       1  MIITQTSHCYMTSLGILFLINILPGTTGQGESRRQEPGDFVKQDIGGLSPKHAPDIPDDS  60
α3  Mouse       1  MIITQMWHFYVTRVVLLLLISILPGTTSQGESRRQEPGDFVKQDIGGLSPKHAPDIPDDS  60
α3  Zebrafish   1  ----------------MAGVFHKLTSCVCCVWILLSLLFNPSSQTGAVTGHREP-IPDDS  43

α3  Human      61  TDNITIFTRILDRLLDGYDNRLRPGLGDAVTEVKTDIYVTSFGPVSDTDMEYTIDVFFRQ  120
α3  Mouse      61  TDNITIFTRILDRLLDGYDNRLRPGLGDAVTEVKTDIYVTSFGPVSDTDMEYTIDVFFRQ  120
α3  Zebrafish  44  KDNITIFTRILDRLLDGYDNRLRPGLGESVTEVRINIYVTSFGPVSDTDMEYTIDVFFRQ  103

α3  Human     121  TWHDERLKFDGPMKILPLNNLLASKIWTPDTFFHNGKKSVAHNMTTPNKLLRLVDNGTLL  180
α3  Mouse     121  TWHDERLKFDGPMKILPLNNLLASKIWTPDTFFHNGKKSVAHNMTTPNKLLRLVDNGTLL  180
α3  Zebrafish 104  SWRDERLKFDGPMQVLPLNNLLASKIWTPDTFFHNGKKSVAHNMTTPNKLLRLVDNGTLL  163

α3  Human     181  YTMRLTIHAECPMHLEDFPMDVHACPLKFGSYAYTTAEVVYSWTLGKNKSVEVAQDGSRL  240
α3  Mouse     181  YTMRLTIHAECPMHLEDFPMDVHACPLKFGSYAYTKAEVIYSWTLGKNKSVEVAQDGSRL  240
α3  Zebrafish 164  YTMRLTIHAECPMHLEDFPMDAHACPLKFGSYAYTNNEVVYIWTADDEKSVSVAPDGSRL  223
                                                              M1
α3  Human     241  NQYDLLGHVVGTEIIRSSTGEYVVMTTHFHLKRKIGYFVIQTYLPCIMTVILSQVSFWLN  300
α3  Mouse     241  NQYDLLGHVVGTEIIRSSTGEYVVMTTHFHLKRKIGYFVIQTYLPCIMTVILSQVSFWLN  300
α3  Zebrafish 224  NQYDLLGHVIGKETISSSTGEYVVMTTYFHLKRKIGYFVIQTYLPCIMTVILSQVSFWLN  283
                              M2                              M3
α3  Human     301  RESVPARTVFGVTTVLTMTTLSISARNSLPKVAYATAMDWFIAVCYAFVFSALIEFATVN  360
α3  Mouse     301  RESVPARTVFGVTTVLTMTTLSISARNSLPKVAYATAMDWFIAVCYAFVFSALIEFATVN  360
α3  Zebrafish 284  RESVPARTVFGVTTVLTMTTLSISARNSLPKVAYATAMDWFMAVCYAFVFSALIEFATVN  343

α3  Human     361  YFTKRSWAWEGKKVPEALEMKKKTPAAPAKKTSTTFNIVGTTYPINLAKDTEFSTISKGA  420
α3  Mouse     361  YFTKRSWAWEGKKVPEALEMKKKTPAAPTKK-NTTFNIVGTTYPINLAKDTEFSTISKSA  419
α3  Zebrafish 344  YFTKRSWAWDGQK--EAQEMRRRESASFSKKTNNTFNIVGTTYSMSVVKDPGLTTISKSA  401
                                                                     M4
α3  Human     421  A-PSASSTPTIIASPKATYVQDS--PTETKTYNSVSKVDKISRIIFPVLFAIFNLVYWAT  477
α3  Mouse     420  AAPSASSTPTAIASPKATYVQDS--PAETKTYNSVSKVDKISRIIFPVLFAIFNLVYWAT  477
α3  Zebrafish 402  T-PTTSTPPQPVREVRLPKMDGEYVPEGRKSYNRVSKVDKISRIIFPVLFFIFNLGYWAT  460

α3  Human     478  YVNRESAIKGMIRKQ                                              492
α3  Mouse     478  YVNRESAIKGMIRKQ                                              492
α3  Zebrafish 461  YVNRKPTIERSSPPK                                              475
```

Sadamitsu et al. Supplementary Figure 3

GABA$_A$ receptor subunit α4

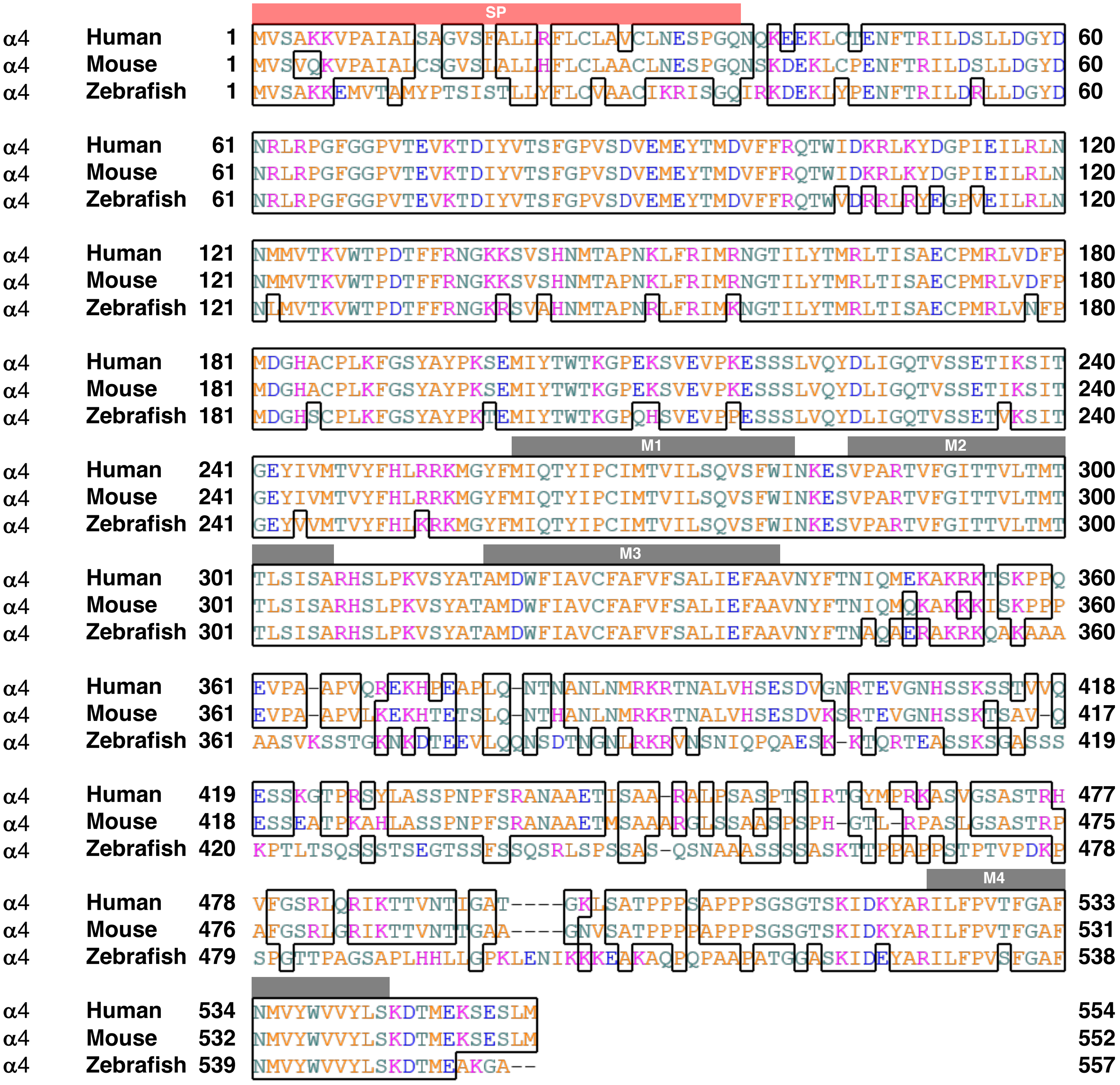

Sadamitsu et al. Supplementary Figure 4

GABA$_A$ receptor subunit α5

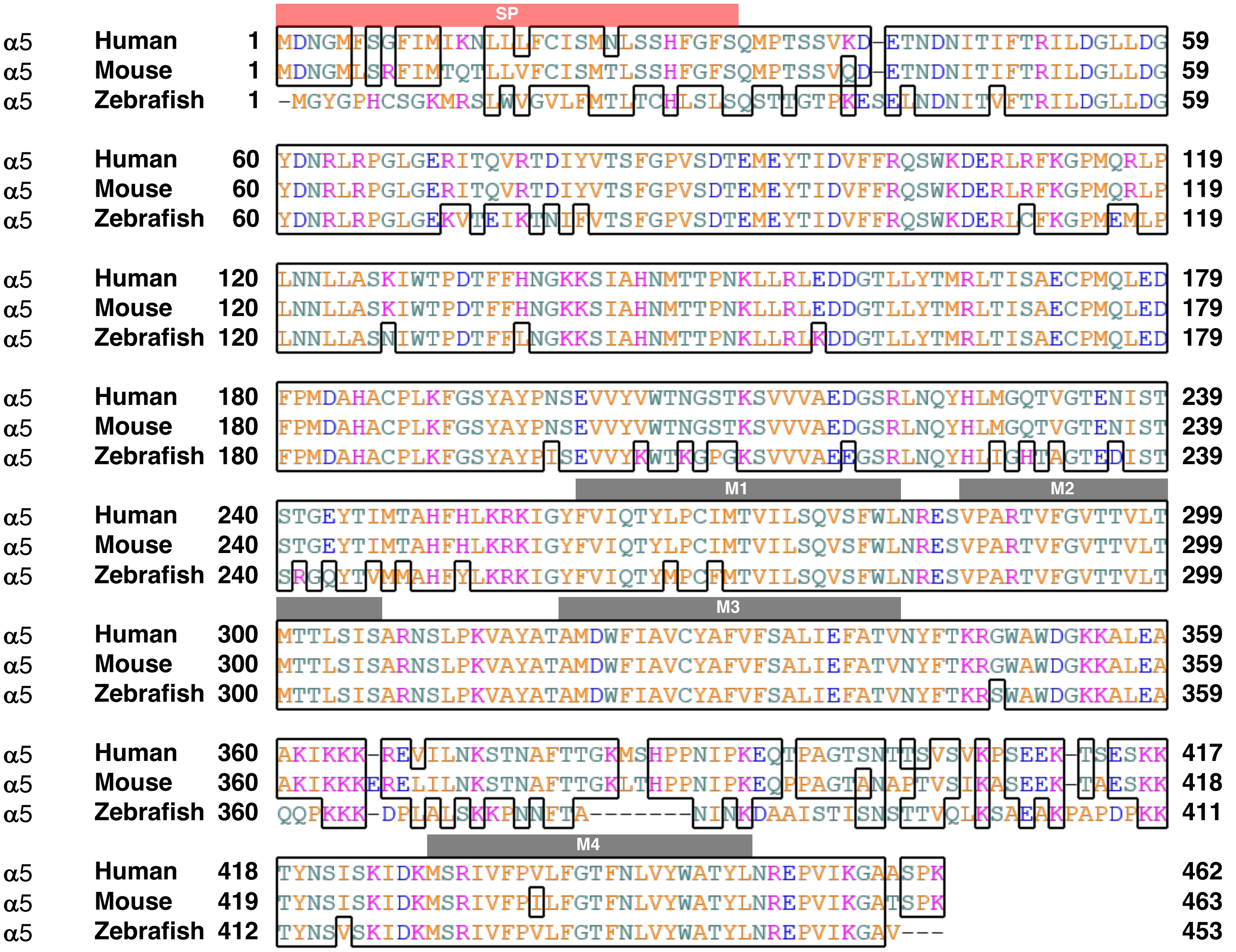

Sadamitsu et al. Supplementary Figure 5

GABA$_A$ receptor subunit α6

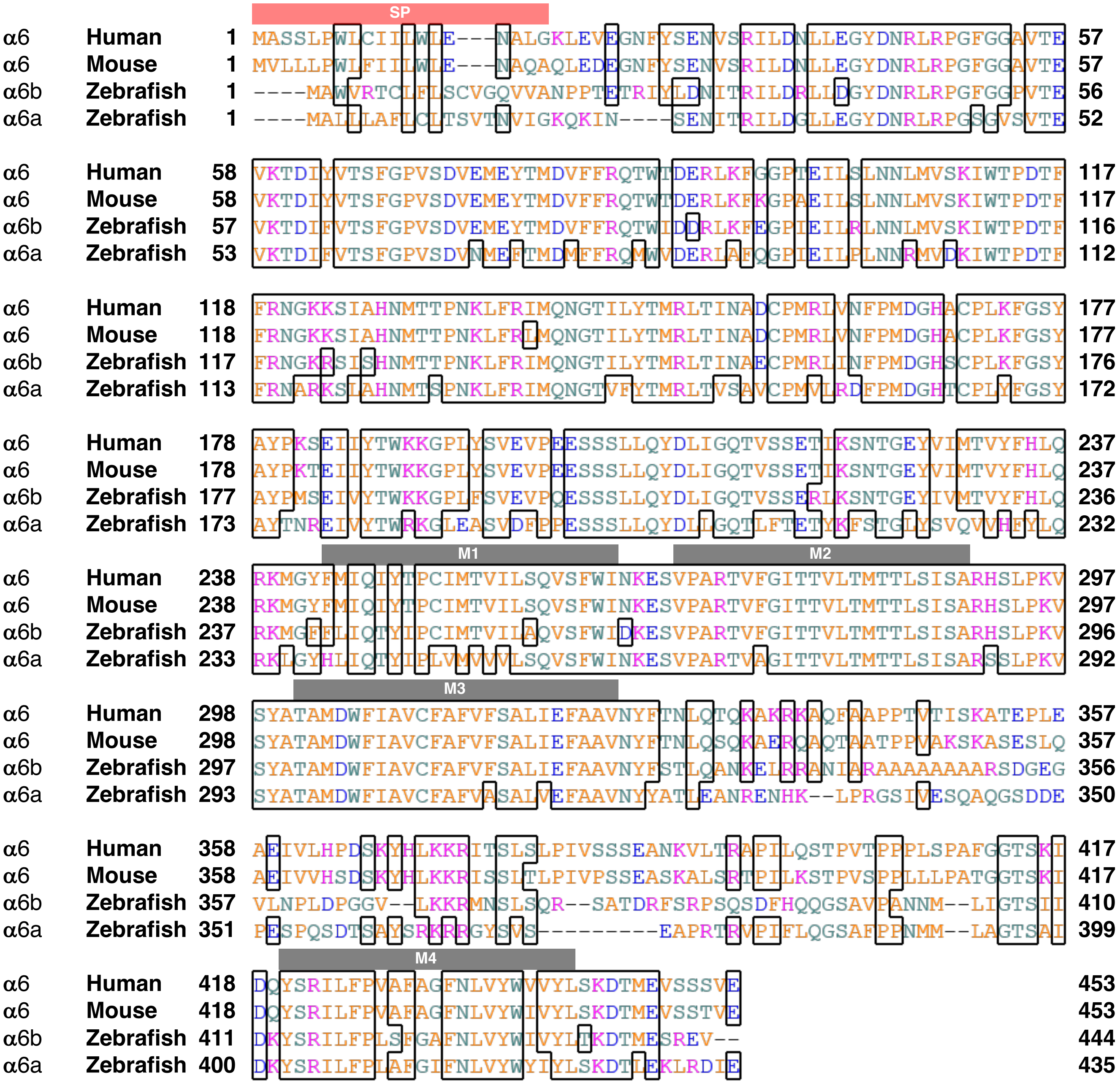

Sadamitsu et al. Supplementary Figure 6

GABA$_A$ receptor subunit β1

```
                            SP
β1  Human       1  --------MWTVQNRESLGLLSFPVMITMVCCAHSTNEPSNMSYVKETVDRLLKGYDIRL  52
β1  Mouse       1  --------MWTVQNRESLGLLSFPVMVAMVCCAHSSNEPSNMSYVKETVDRLLKGYDIRL  52
β1  Zebrafish   1  MMRGMRIAGDQWRAGIFCLLVLVALTLGRSPPAHSVNEPSNMSYVKVTVDKLLKGYDIRL  60

β1  Human      53  RPDFGGPPVDVGMRIDVASIDMVSEVNMDYTLTMYFQQSWKDKRLSYSGIPLNLTLDNRV  112
β1  Mouse      53  RPDFGGPPVDVGMRIDVASIDMVSEVNMDYTLTMYFQQSWKDKRLSYSGIPLNLTLDNRV  112
β1  Zebrafish  61  RPDFGGPPVDVGMSIDISSIDMVSEVNMDYTITMYFQQSWRDKRLSYTGIPLNLTLDNRV  120

β1  Human     113  ADQLWVPDTYFLNDKKSFVHGVTVKNRMIRLHPDGTVLYGLRITTTAACMMDLRRYPLDE  172
β1  Mouse     113  ADQLWVPDTYFLNDKKSFVHGVTVKNRMIRLHPDGTVLYGLRITTTAACMMDLRRYPLDE  172
β1  Zebrafish 121  ADQLWVPDTYFINDKKSFVHGVTVKNRMIRLHPDGTVLYGLRITTTAACMMDLRRYPLDE  180

β1  Human     173  QNCTLEIESYGYTTDDIEFYWNGGEGAVTGVNKIELPQFSIVDYKMVSKKVEFTTGAYPR  232
β1  Mouse     173  QNCTLEIESYGYTTDDIEFYWNGGEGAVTGVNKIELPQFSIVDYKMVSKKVEFTTGAYPR  232
β1  Zebrafish 181  QNCTLEIESYGYTTDDIEFYWQGG-SSVTGVDNIELPQFSIIDYKTLSKKVVFATGSYPR  239
                                                     M1                M2
β1  Human     233  LSLSFRLKRNIGYFILQTYMPSTLITILSWVSFWINYDASAARVALGITTVLTMTTISTH  292
β1  Mouse     233  LSLSFRLKRNIGYFILQTYMPSTLITILSWVSFWINYDASAARVALGITTVLTMTTISTH  292
β1  Zebrafish 240  LSLSFKLKRNIGYFILQTYMPSTLITILSWVSFWINYDASAARVALGITTVLTMTTINTH  299
                                      M3
β1  Human     293  LRETLPKIPYVKAIDIYLMGCFVFVFLALLEYAFVNYIFFGKGP--QKKGASKQDQSANE  350
β1  Mouse     293  LRETLPKIPYVKAIDIYLMGCFVFVFLALLEYAFVNYIFFGKGP--QKKGASKQDQSANE  350
β1  Zebrafish 300  LRETLPKIPYVKAIDIYLMGCFVFVFLALLEYAFVNYIFFGRGPHLQKKVAEKAAKSNNE  359

β1  Human     351  KNKLEMNKVQVDAHGNILLSTLEIRNETSGSEVLTSVSDPKATMYSYDSASIQYRKPLSS  410
β1  Mouse     351  KNRLEMNKVQVDAHGNILLSTLEIRNETSGSEVLTGVSDPKATMYSYDSASIQYRKPLSS  410
β1  Zebrafish 360  KSRLESNKVQVDVHGSIPLTNLDLR--LSGAEMLGALGDPRNTMFSYDSASIQYRKPLTG  417
                                                                       M4
β1  Human     411  REAYGR---ALDRHGVPSKGRIRRRASQLKVKIPDLTDVNSIDKWSRMFFPITFSLFNVV  467
β1  Mouse     411  REGFGR---GLDRHGVPGKGRIRRRASQLKVKIPDLTDVNSIDKWSRMFFPITFSLFNVV  467
β1  Zebrafish 418  RDLYGRPTASIERPLAPKKSRLRRRAAQLKVKIPDLTDVNAIDKWSRVIFPITYTFFNLV  477

β1  Human     468  YWLYYVH                                                       474
β1  Mouse     468  YWLYYVH                                                       474
β1  Zebrafish 478  YWLYYVH                                                       484
```

Sadamitsu et al. Supplementary Figure 7

GABA$_A$ receptor subunit β2

```
                          SP
β2  Human        1  MWRVRKRGYFGIWSFPLIIAAVCAQSVNDPSNMSLVKETVDRLLKGYDIRLRPDFGGPPV  60
β2  Mouse        1  MWRVRKRGYFGIWSFPLIIAAVCAQSVNDPSNMSLVKETVDRLLKGYDIRLRPDFGGPPV  60
β2  Zebrafish    1  -MESIGKTPHIFLLCPLIVAVACAQSIRDPSNMPVVKDTVDRLMKGYDIRLRPDFGGAPV  59

β2  Human       61  AVGMNIDIASIDMVSEVNMDYTLTMYFQQAWRDKRLSYNVIPLNLTLDNRVADQLWVPDT  120
β2  Mouse       61  AVGMNIDIASIDMVSEVNMDYTLTMYFQQAWRDKRLSYNVIPLNLTLDNRVADQLWVPDT  120
β2  Zebrafish   60  AVGMNIDIASIDMVSEVNMDYTLTMYFQQAWRDKRLSYSEIPLNLTLDNRVADQLWVPDT  119

β2  Human      121  YFLNDKKSFVHGVTVKNRMIRLHPDGTVLYGLRITTTAACMMDLRRYPLDEQNCTLEIES  180
β2  Mouse      121  YFLNDKKSFVHGVTVKNRMIRLHPDGTVLYGLRITTTAACMMDLRRYPLDEQNCTLEIES  180
β2  Zebrafish  120  YFLNDKKSFVHGVTVKNRMIRLHPDGTVLYGLRITTTAACMMDLRRYPLDEQNCTLEIES  179

β2  Human      181  YGYTTDDIEFYWRGDDNAVTGVTKIELPQFSIVDYKLITKKVVFSTGSYPRLSLSFKLKR  240
β2  Mouse      181  YGYTTDDIEFYWRGDDNAVTGVTKIELPQFSIVDYKLITKKVVFSTGSYPRLSLSFKLKR  240
β2  Zebrafish  180  YGYTTDDIEFYWRGGDGAVSGVERIELPQFSIVDYKLISKNVVFSTGSYPRLSLSFKLKR  239

                          M1                    M2
β2  Human      241  NIGYFILQTYMPSILITILSWVSFWINYDASAARVALGITTVLTMTTINTHLRETLPKIP  300
β2  Mouse      241  NIGYFILQTYMPSILITILSWVSFWINYDASAARVALGITTVLTMTTINTHLRETLPKIP  300
β2  Zebrafish  240  NIGYFILQTYMPSILITILSWVSFWINYDASAARVALGITTVLTMTTINTHLRETLPKIP  299

                          M3
β2  Human      301  YVKAIDMYLMGCFVFVFMALLEYALVNYIFFGRGPQRQKKAAEKAASANNEKMRLDVNKM  360
β2  Mouse      301  YVKAIDMYLMGCFVFVFMALLEYALVNYIFFGRGPQRQKKAAEKAANANNEKMRLDVNKM  360
β2  Zebrafish  300  YVKAIDMYLMGCFVFVFLALLEYAFVNYIFFGRGPQRQKKAAEKAATANNEKLRPDPNKM  359

β2  Human      361  DPHENILLSTLEIKNEMATSEAVMGLGDPRSTMLAYDASSIQYRKAGLPRHSFGRNALER  420
β2  Mouse      361  DPHENILLSTLEIKNEMATSEAVMGLGDPRSTMLAYDASSIQYRKAGLPRHSFGRNALER  420
β2  Zebrafish  360  TP-DDIRLTTVEMKNEMGPSDLSRGLGDPRSTMLAYDSSTLQYRRAAMARQNYGHSALER  418

                                                      M4
β2  Human      421  HVAQKKSRLRRRASQLKITIPDLTDVNAIDRWSRIFFPVVFSFFNIVYWLYYVN        474
β2  Mouse      421  HVAQKKSRLRRRASQLKITIPDLTDVNAIDRWSRIFFPVVFSFFNIVYWLYYVN        474
β2  Zebrafish  419  HATQKKSRLRRRASQLKVNIPDLSDVNSIDKWSRMIFPTLFSFFNIVYWLYYVH        472
```

Sadamitsu et al. Supplementary Figure 8

GABA$_A$ receptor subunit β3/4

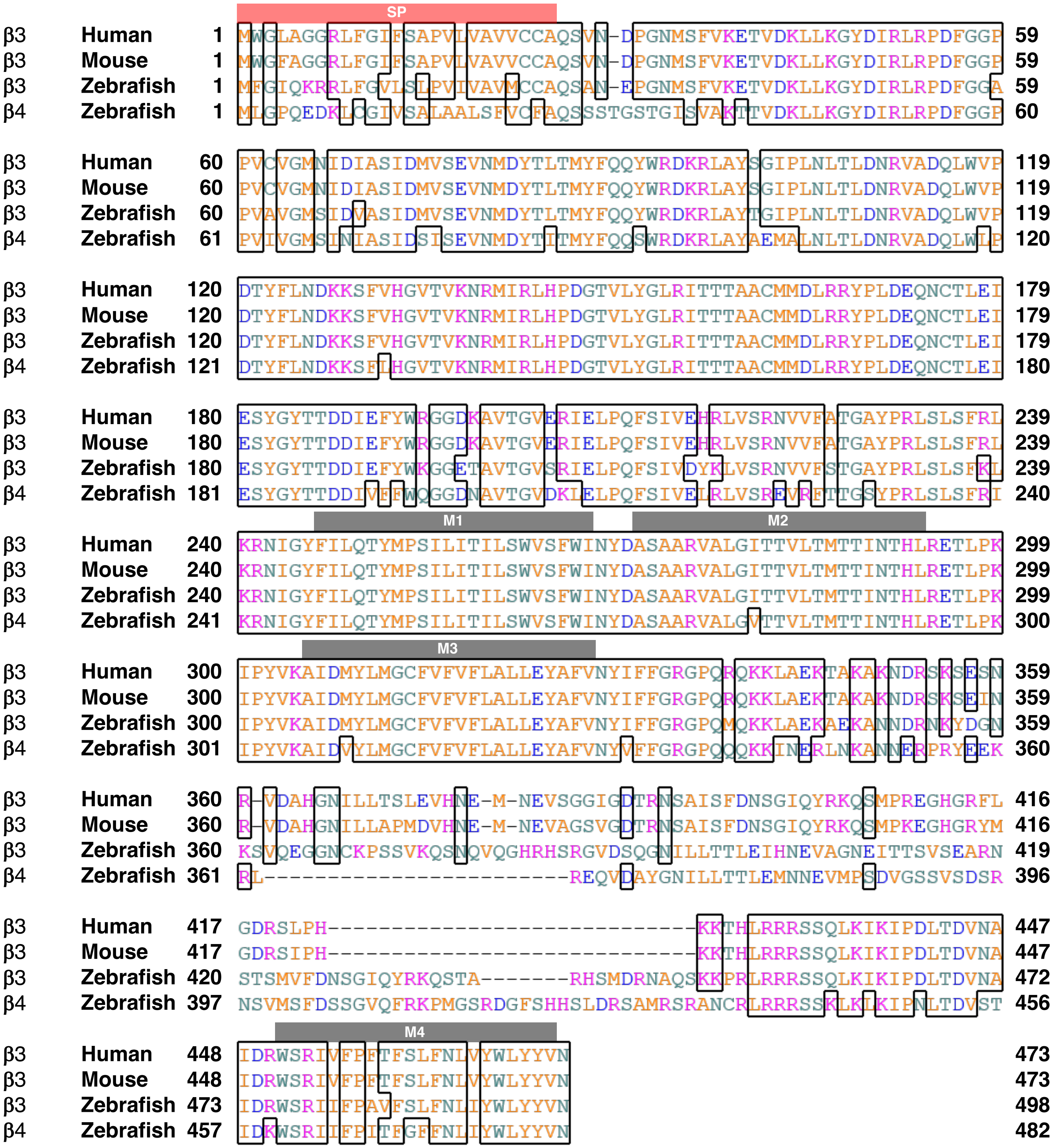

Sadamitsu et al. Supplementary Figure 9

GABA$_A$ receptor subunit γ1

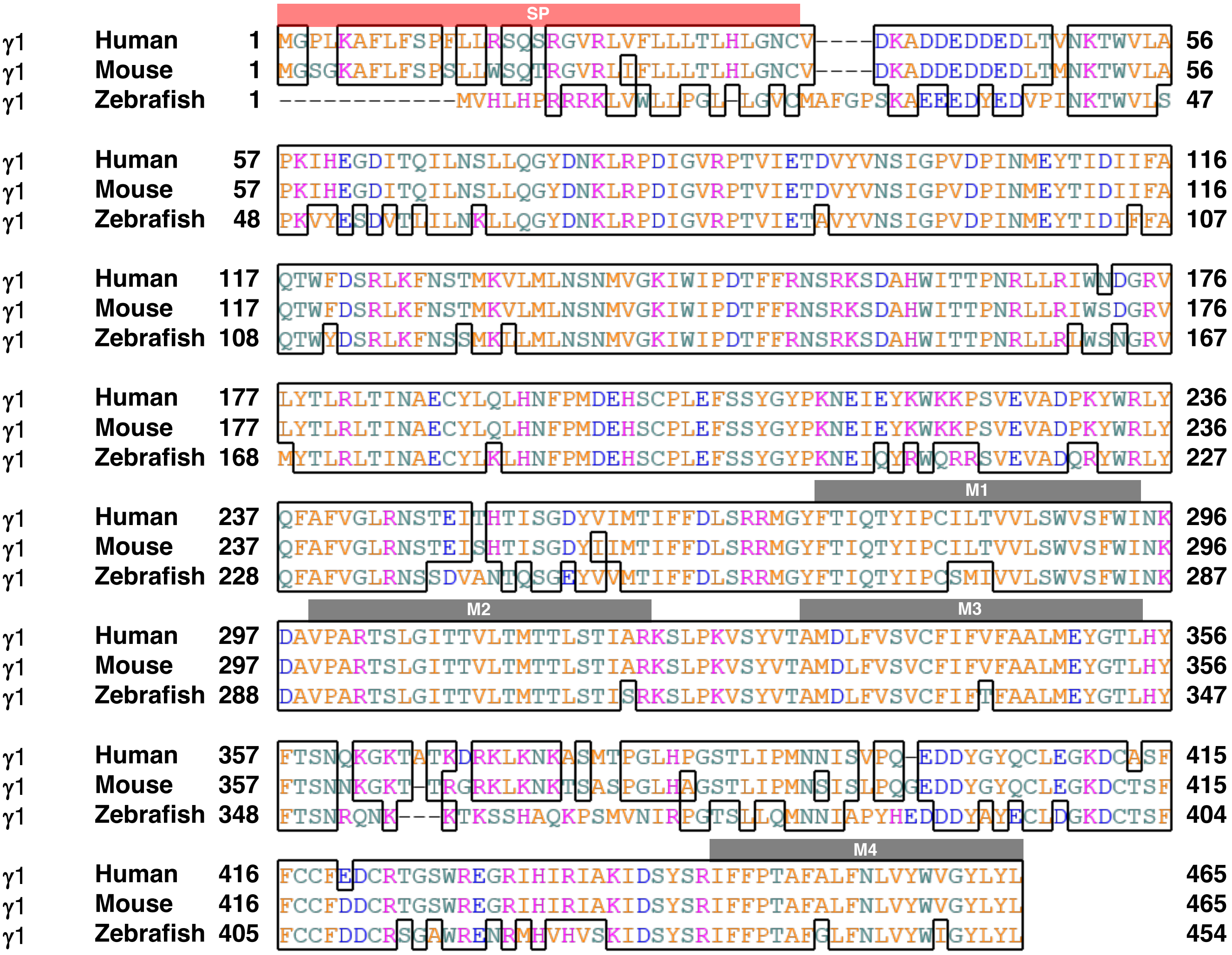

Sadamitsu et al. Supplementary Figure 10

GABA$_A$ receptor subunit γ2

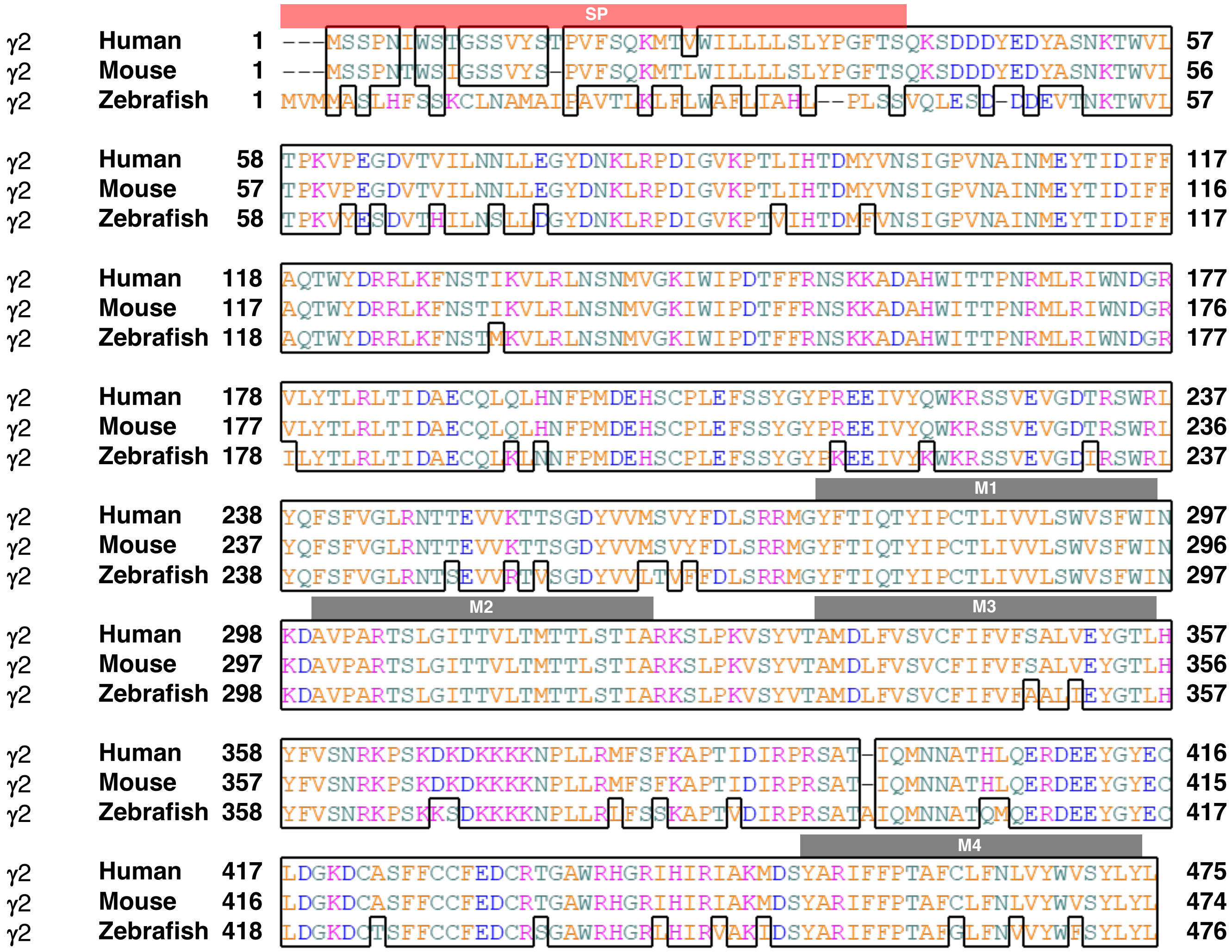

Sadamitsu et al. Supplementary Figure 11

GABA$_A$ receptor subunit γ3

```
                                SP
γ3  Human       1  MAPKLLLLLCLFSGLHARSRKVEEDEYEDSSSNQKWVLAPKSQDTDVTLILNKLLREYDK   60
γ3  Mouse       1  MAAKLLLLLCLFSGLHARSRRVEEDENEDSPSNQKWVLAPKSQDTDVTLILNKLLREYDK   60
γ3  Zebrafish   1  MTTKLFLYFLLLSVFRACSPTFASDDDEYDDVTVNQMLAPKTHETDATQILNNLLKEYDK   60

γ3  Human      61  KLRPDIGIKPTVIDVDIYVNSIGPVSSINMEYQIDIFFAQTWTDSRLRFNSTMKILTLNS  120
γ3  Mouse      61  KLRPDIGIKPTVIDVDIYVNSIGPVSSINMEYQIDIFFAQTWTDSRLRFNSTMKILTLNS  120
γ3  Zebrafish  61  KLRPDIGVKPTVIDVDIYVNSIGPVSSINMEYQIDIIFAQTWTDSRLRYNSTMKILTLNS  120

γ3  Human     121  NMVGLIWIPDTIFRNSKTAEAHWITTPNQLLRIWNDGKILYTLRLTINAECQLQLHNFPM  180
γ3  Mouse     121  NMVGLIWIPDTIFRNSKTAEAHWITTPNQLLRIWNDGKILYTLRLTINAECQLQLHNFPM  180
γ3  Zebrafish 121  NMVGLIWLPDTIFRNSKSADSHWITTPNQLLRIWNDGKILYTLRLTINAECQLQLHNFPM  180

γ3  Human     181  DEHSCPLIFSSYGYPKEEMIYRWRKNSVEAADQKSWRLYQFDFMGLRNTTEIVTTSAGDY  240
γ3  Mouse     181  DAHACPLTFSSYGYPKEEMIYRWRKNSVEAADQKSWRLYQFDFMGLRNTTEIVTTSAGDY  240
γ3  Zebrafish 181  DEHSCPLIFSSYGYPRDEMIYKWRRNSVQAADQKSWRLYQFDFMGLRNTTDVIKTTAGDY  240
                                        M1                     M2
γ3  Human     241  VVMTIYFELSRRMGYFTIQTYIPCILTVVLSWVSFWIKKDATPARTAL------------  288
γ3  Mouse     241  VVMTIYFELSRRMGYFTIQTYIPCILTVVLSWVSFWIKKDATPARTTL------------  288
γ3  Zebrafish 241  VVMTVYFDLSRRMGYFTIQTYIPCILTVVLSWVSFWIKKDATPARTALVSCFKSMSCPGK  300
                                                   M3
γ3  Human     289  -----GITTVLTMTTLSTIARKSLPRVSYVTAMDLFVTVCFLFVFAALMEYATLNYYS-S  342
γ3  Mouse     289  -----GITTVLTMTTLSTIARKSLPRVSYVTAMDLFVTVCFLFVFAALMEYATLNYYS-S  342
γ3  Zebrafish 301  CHCCQGITTVLTMTTLSTVARTSLPRVSYVTAMDLFVTVCFLFVFAALMEYATLNYYSYS  360

γ3  Human     343  CRKPTTTKKTTSLLHPDSSRWIPERISLQAPSNYSLLDMRPPPTAMITLNNSVYWQEFED  402
γ3  Mouse     343  CRKPTIRKKKTSLLHPDSTRWIPDRISLQAPSNYSLLDMRPPPPVMITLNNSMYWQEFED  402
γ3  Zebrafish 361  ARRPTCNKTKRS--------------------NYSVLDVGPPPTV-ITLNNSMYWQEFDD  399
                                                                      M4
γ3  Human     403  TCVYECLDGKDCQSFFCCYEECKSGSWRKGRIHIDILELDSYSRVFFPTSFLLFNLVYWV  462
γ3  Mouse     403  TCVYECLDGKDCQSFFCCYEECKSGSWRRGRIHIDVSELDSYSRVFFPTSFLLFNLVYWV  462
γ3  Zebrafish 400  ACVYECLDGKDCQSFFCCYEECKDGAWRKGRVHIDILELDAYSRVFFPTSFLLFNVVYWV  459

γ3  Human     463  GYLYL                                                         467
γ3  Mouse     463  GYLYL                                                         467
γ3  Zebrafish 460  GYLYL                                                         464
```

Sadamitsu et al. Supplementary Figure 12

GABA$_A$ receptor subunit δ

```
δ  Human       1  MDAPARLLAPLLLLCAQQLR-GTRAMNDIGDYVGSNLEISWLPNLDGLIAGYARNFRPGI   59
δ  Mouse       1  MDVLGWLLLPLLLLC-TQPHHGARAMNDIGDYVGSNLEISWLPNLDGLMEGYARNFRPGI   59
δ  Zebrafish   1  MDALSLTVKLFWLVLIGGDRRSRAMLSDIGDYVGTDIEISWLPNLDDLMKGYARNFRPGI   60

δ  Human      60  GGPPVNVALALEVASIDHISEANMEYTMTVFLHQSWRDSRLSYNHTNETLGLDSRFVDKL  119
δ  Mouse      60  GGAPVNVALALEVASIDHISEANMEYTMTVFLHQSWRDSRLSYNHTNETLGLDSRFVDKL  119
δ  Zebrafish  61  GGPPVNVAMAIEVASIDHISEANMEYTMTVFLRQSWRDDRLSYNHTNKTLGLDSRFVDKL  120

δ  Human     120  WLPDTFIVNAKSAWFHDVTVENKLIRLQPDGVILYSIRITSTVACDMDLAKYPMDEQECM  179
δ  Mouse     120  WLPDTFIVNAKSAWFHDVTVENKLIRLQPDGVILYSIRITSTVACDMDLAKYPLDEQECM  179
δ  Zebrafish 121  WLPDTFIVNAKSAWFHDVTVENKLIRLQPDGVILYSSRITSTVACDMDLTKYPMDEQECM  180

δ  Human     180  LDLESYGYSSEDIVYYWSESQEHIHGLDKLQLAQFTITSYRFTTELMNFKSAGQFPRLSL  239
δ  Mouse     180  LDLESYGYSSEDIVYYWSENQEQIHGLDRLQLAQFTITSYRFTTELMNFKSAGQFPRLSL  239
δ  Zebrafish 181  LDLESYGYSSEDIVYHWSESQKLIHGLDKLELSQFTITDYRFVTEMMNFKSAGRFPRLSL  240

δ  Human     240  HFHLRRNRGVYIIQSYMPSVLLVAMSWVSFWISQAAVPARVSLGITTVLTMTTLMVSARS  299
δ  Mouse     240  HFQLRRNRGVYIIQSYMPSVLLVAMSWVSFWISQAAVPARVSLGITTVLTMTTLMVSARS  299
δ  Zebrafish 241  RFQLRRNRGVYIIQSYMPSILLVAMSWVSFWISQSAVPARVSLGITTVLTMTTLMVSARS  300

δ  Human     300  SLPRASAIKALDVYFWICYVFVFAALVEYAFAHFNADYRKKQKAKVKVSRPRAEM--DVR  357
δ  Mouse     300  SLPRASAIKALDVYFWICYVFVFAALVEYAFAHFNADYRKKRKAKVKVTKPRAEM--DVR  357
δ  Zebrafish 301  SLPRASAIKALDVYFWICYVFVFAALIEYAFAHYNADYSKKEKAKVKTNKNAESMVKNGK  360

δ  Human     358  NAIVLFSLSAAGVTQELAISRRQRRVPGNLMGSYRSVGVETGETKKEGAARSGGQGGIRA  417
δ  Mouse     358  NAIVLFSLSAAGVSQELAISRRQGRVPGNLMGSYRSVEVEA---KKEGGSRPGGPGGIRS  414
δ  Zebrafish 361  QAMVLFSLSVAGMNQGLLISNRQSRSHRASDAAAAAADPHEETETRSATSRRAQKESSEE  420

δ  Human     418  RL----RPIDADTIDIYARAVFPAAFAAVNVIYWAAYAM                       452
δ  Mouse     415  RL----KPIDADTIDIYARAVFPAAFAAVNIIYWAAYTM                       449
δ  Zebrafish 421  KKCCKCKPIDADTIDIYARAVFPFTFAVVNVIYWVAYTM                       459
```

Sadamitsu et al. Supplementary Figure 13

$GABA_A$ receptor subunit π

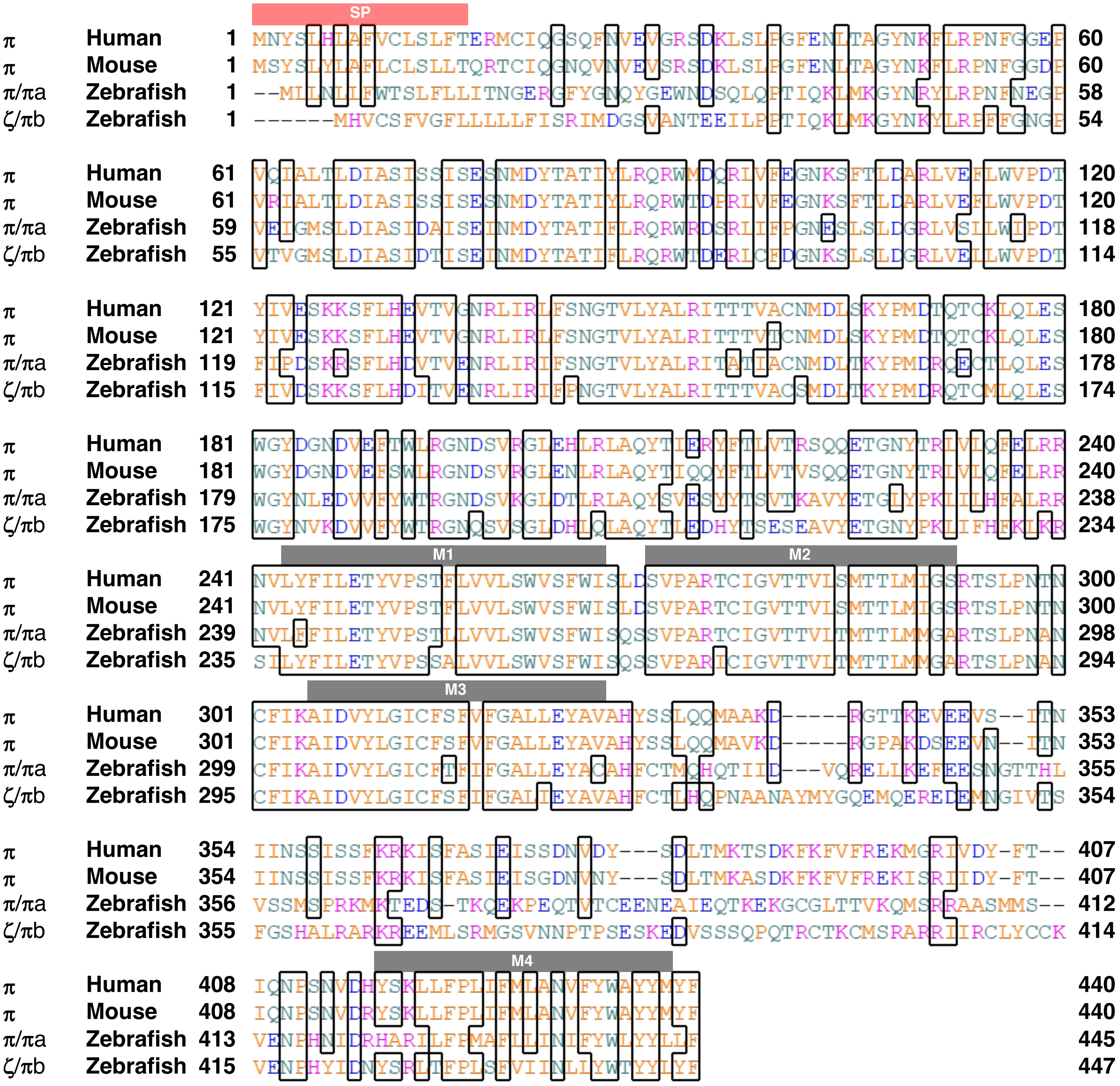

Sadamitsu et al. Supplementary Figure 14

GABA$_A$ receptor subunit ρ1

```
                            SP
ρ1  Human       1  MLAVPNMRFGIFLLWWGWVLATESRMHWPGREVHEMSKKG-RPQRQRREVHEDAHKQVSP  59
ρ1  Mouse       1  MLAVQNMKFGIFLLWWGWVLAAESTAHWPGREVHEPSRKGSRPQRQRRGAHDDAHKQGSP  60
ρ1  Zebrafish   1  ----------------MHADVCFLLCACFLCTGALQSCSKSRSKRETTRAERDLHKPGST  44

ρ1  Human      60  ILRRSPDITKSPLTKSEQLLRIDDHDFSMRPGFGGPAIPVGVDVQVESLDSISEVDMDFT  119
ρ1  Mouse      61  ILRRSSDITKSPLTKSEQLLRIDDHDFSMRPGFGGPAIPVGVDVQVESLDSISEVDMDFT  120
ρ1  Zebrafish  45  ILMRSPDVTKAFVTKSEQLIKIDDHDFTMRPGFGGPAIPVGVDVQLESLDTISEVDMDFT  104

ρ1  Human     120  MTLYLRHYWKDERLSFPSTNNLSMTFDGRLVKKIWVPDMFFVHSKRSFIHDTTTDNVMLR  179
ρ1  Mouse     121  MTLYLRHYWKDERLSFPSSNNLSMTFDGRLVKKIWVPDMFFVHSKRSFIHDTTTDNVMLR  180
ρ1  Zebrafish 105  MTLYLRHYWKDARLSFPSNTNQSMTFDGRLVKKIWVPDIFFVHSKRSFIHDTTTENVMLR  164

ρ1  Human     180  VQPDGKVLYSLRVTVTAMCNMDFSRFPLDTQTCSLEIESYAYTEDDLMLYWKKGNDSLKT  239
ρ1  Mouse     181  VQPDGKVLYSLRVTVTAMCNMDFSRFPLDTQTCSLEIESYAYTEDDLMLYWKKGNDSLKT  240
ρ1  Zebrafish 165  VHPDGKVLYSLRVTVTSMCNMDLSHFPLDTQTCSLEIESYAYTDDDLMLYWKRGNKSLQT  224

                                                                       M1
ρ1  Human     240  DERISLSQFLIQEFHTTTKLAFYSSTGWYNRLYINFTLRRHIFFFLLQTYFPATLMVMLS  299
ρ1  Mouse     241  DERISLSQFLIQEFHTTTKLAFYSSTGWYNRLYINFTLRRHIFFFLLQTYFPATLMVMLS  300
ρ1  Zebrafish 225  DDKISLSQFLIQEFHTTTKLAFYSSTGWYNRLYINFTLRRHIFFFLLQTYFPATLMVMLS  284

                                      M2                           M3
ρ1  Human     300  WVSFWIDRRAVPARVPLGITTVLTMSTIITGVNASMPRVSYIKAVDIYLWVSFVFVFLSV  359
ρ1  Mouse     301  WVSFWIDRRAVPARVPLGITTVLTMSTIITGVNASMPRVSYIKAVDIYLWVSFVFVFLSV  360
ρ1  Zebrafish 285  WVSFWIDRRAVPARVPLGITTVLTMSTIITGVNASMPRVSYIKAVDIYLWVSFVFVFLSV  344

ρ1  Human     360  LEYAAVNYLTTVQERKEQKLREK-LPCTSGLPPPRTAMLDGNYSDGEVNDLDNY-MPE-N  416
ρ1  Mouse     361  LEYAAVNYLTTVQERKERKLREK-ISCTCGLPQPRGVMLDSSYSDGEVNDLGGY-MPE-N  417
ρ1  Zebrafish 345  IEYAAVNYLSTIQERKERSQNSRQLPCTCGMTHPGQMMMSSSYSEMDMMTTGNYGMSELS  404

                                                                    M4
ρ1  Human     417  GEKPDRMMVQLTLASERSSPQRKSQRSSYVSMRIDTHAIDKYSRIIFPAAYILFNLIYWS  476
ρ1  Mouse     418  GEKPDRMMVQLTLASERGSPQRKGQRGSYVSMRINTHAIDKYSRIIFPAAYILFNLIYWS  477
ρ1  Zebrafish 405  DTKQEQFLVHLVMDNEQGA-QRPAVTTS-STLNIDTHAIDKYSRVIFPGAYTLFNLIYWS  462

ρ1  Human     477  IFS-                                                          479
ρ1  Mouse     478  IFS-                                                          480
ρ1  Zebrafish 463  IYSQ                                                          466
```

Sadamitsu et al. Supplementary Figure 15

GABA$_A$ receptor subunit ρ2

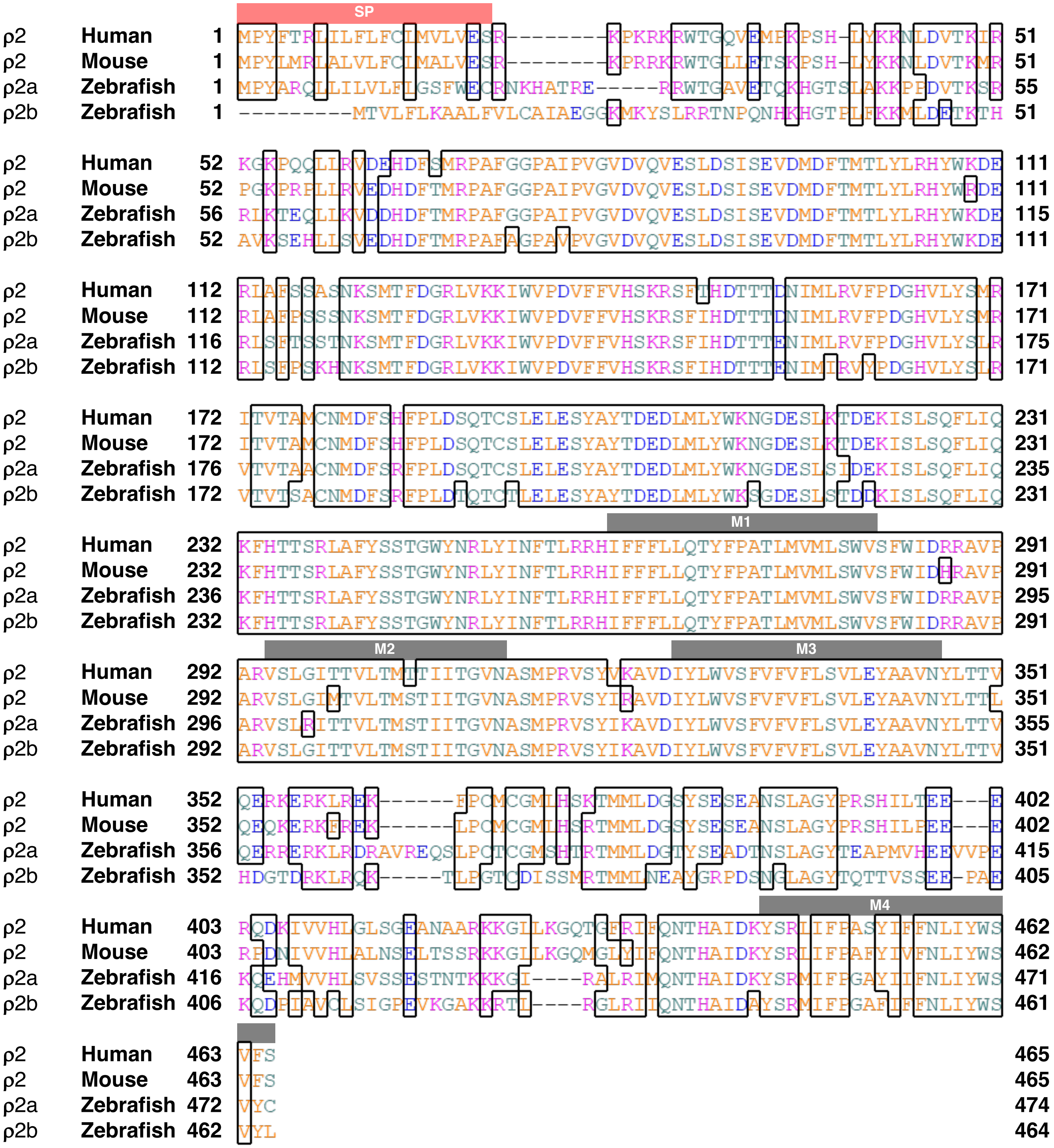

Sadamitsu et al. Supplementary Figure 16

GABA$_A$ receptor subunit ρ3

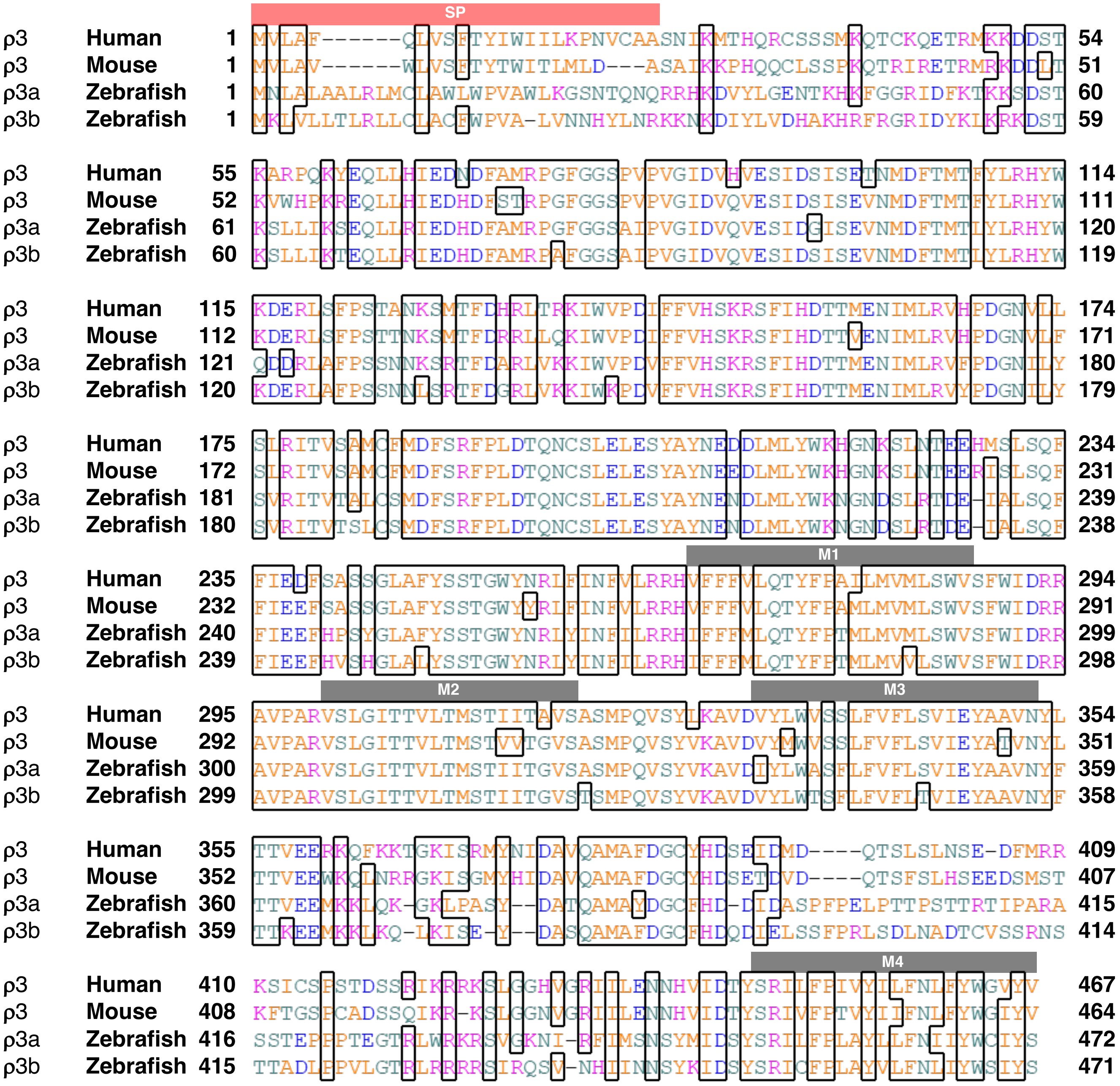

Sadamitsu et al. Supplementary Figure 17

	α1	α2a	α3	α4	α5	α6a	α6b	β1	β2	β3	β4	γ1	γ2	γ3	δ	πa	πb	ρ1	ρ2a	ρ2b	ρ3a	ρ3b
α1	100																					
α2a	67.3	100																				
α3	66.6	64.8	100																			
α4	55.5	56.4	54.7	100																		
α5	64.8	65.0	64.3	54.2	100																	
α6a	54.2	58.4	53.8	49.9	55.8	100																
α6b	54.2	61.5	59.0	59.0	59.2	62.7	100															
β1	49.7	48.6	47.7	46.4	46.2	44.2	50.5	100														
β2	45.9	45.1	45.9	45.6	44.8	41.6	44.4	64.9	100													
β3	50.3	47.0	48.4	48.6	47.6	44.7	49.1	60.8	60.8	100												
β4	47.7	48.2	49.1	49.1	47.2	44.4	49.7	65.9	63.3	66.3	100											
γ1	51.7	53.8	51.6	49.8	51.4	49.5	54.6	49.7	46.1	48.7	47.4	100										
γ2	51.4	54.1	55.2	50.4	51.6	49.5	53.7	50.8	48.8	46.9	47.4	67.7	100									
γ3	48.1	50.6	48.3	47.3	50.0	48.4	52.6	48.9	44.5	48.4	45.4	61.7	60.8	100								
δ	48.7	48.1	49.0	44.4	48.7	47.4	46.4	53.3	50.0	51.7	52.2	50.3	49.3	49.0	100							
πa	45.5	46.1	46.5	45.2	44.7	47.0	48.4	52.4	40.7	48.4	50.6	47.9	45.4	47.0	53.3	100						
πb	45.5	48.7	45.9	44.1	48.3	44.7	48.0	50.5	48.4	51.4	51.0	47.8	47.6	48.5	55.9	63.8	100					
ρ1	47.1	48.3	48.3	45.6	50.1	48.5	48.3	51.0	51.1	50.7	47.9	47.8	51.1	45.9	51.6	51.3	51.1	100				
ρ2a	48.9	48.0	46.8	43.9	46.4	45.3	47.2	50.2	50.5	50.9	52.5	47.4	48.2	47.1	51.6	50.8	49.4	67.7	100			
ρ2b	50.0	46.8	48.9	45.9	47.7	48.4	48.2	51.3	50.9	50.1	52.1	45.0	47.9	46.7	50.3	52.1	49.8	72.0	72.1	100		
ρ3a	48.8	46.3	47.1	43.8	46.6	46.4	50.2	49.5	47.9	48.5	49.3	47.2	48.5	47.3	50.3	48.7	52.0	62.1	62.6	64.1	100	
ρ3b	48.4	47.4	48.2	44.8	48.3	46.4	46.2	47.9	49.6	47.8	52.1	48.3	44.7	46.1	50.7	49.6	51.8	64.0	64.1	64.5	75.0	100

Sadamitsu et al. Supplementary Figure 18
